# Evolution and functional divergence of glycosyltransferase genes shaped the quality and cold tolerance of tea plants

**DOI:** 10.1093/plcell/koae268

**Published:** 2024-10-04

**Authors:** Jingming Wang, Yutong Hu, Danyang Guo, Ting Gao, Tianqi Liu, Jieyang Jin, Mingyue Zhao, Keke Yu, Wei Tong, Honghua Ge, Yuting Pan, Mengting Zhang, Mengqian Lu, Tingting Jing, Wenkai Du, Xiaoyan Tang, Chenjie Zhao, Wei Zhao, Zhijie Bao, Wilfried Schwab, Enhua Xia, Chuankui Song

**Affiliations:** State Key Laboratory of Tea Plant Biology and Utilization, Anhui Agricultural University, 130 Changjiang Ave W., Hefei, Anhui 230036, People's Republic of China; International Joint Laboratory on Tea Chemistry and Health Effects, Anhui Agricultural University, 130 Changjiang Ave W., Hefei, Anhui 230036, People's Republic of China; State Key Laboratory of Tea Plant Biology and Utilization, Anhui Agricultural University, 130 Changjiang Ave W., Hefei, Anhui 230036, People's Republic of China; International Joint Laboratory on Tea Chemistry and Health Effects, Anhui Agricultural University, 130 Changjiang Ave W., Hefei, Anhui 230036, People's Republic of China; State Key Laboratory of Tea Plant Biology and Utilization, Anhui Agricultural University, 130 Changjiang Ave W., Hefei, Anhui 230036, People's Republic of China; International Joint Laboratory on Tea Chemistry and Health Effects, Anhui Agricultural University, 130 Changjiang Ave W., Hefei, Anhui 230036, People's Republic of China; State Key Laboratory of Tea Plant Biology and Utilization, Anhui Agricultural University, 130 Changjiang Ave W., Hefei, Anhui 230036, People's Republic of China; International Joint Laboratory on Tea Chemistry and Health Effects, Anhui Agricultural University, 130 Changjiang Ave W., Hefei, Anhui 230036, People's Republic of China; Institute of Health Sciences and Technology, Anhui University, 111 Jiulong RD., Hefei, Anhui 230601, People's Republic of China; State Key Laboratory of Tea Plant Biology and Utilization, Anhui Agricultural University, 130 Changjiang Ave W., Hefei, Anhui 230036, People's Republic of China; International Joint Laboratory on Tea Chemistry and Health Effects, Anhui Agricultural University, 130 Changjiang Ave W., Hefei, Anhui 230036, People's Republic of China; State Key Laboratory of Tea Plant Biology and Utilization, Anhui Agricultural University, 130 Changjiang Ave W., Hefei, Anhui 230036, People's Republic of China; International Joint Laboratory on Tea Chemistry and Health Effects, Anhui Agricultural University, 130 Changjiang Ave W., Hefei, Anhui 230036, People's Republic of China; State Key Laboratory of Tea Plant Biology and Utilization, Anhui Agricultural University, 130 Changjiang Ave W., Hefei, Anhui 230036, People's Republic of China; International Joint Laboratory on Tea Chemistry and Health Effects, Anhui Agricultural University, 130 Changjiang Ave W., Hefei, Anhui 230036, People's Republic of China; State Key Laboratory of Tea Plant Biology and Utilization, Anhui Agricultural University, 130 Changjiang Ave W., Hefei, Anhui 230036, People's Republic of China; International Joint Laboratory on Tea Chemistry and Health Effects, Anhui Agricultural University, 130 Changjiang Ave W., Hefei, Anhui 230036, People's Republic of China; Institute of Health Sciences and Technology, Anhui University, 111 Jiulong RD., Hefei, Anhui 230601, People's Republic of China; State Key Laboratory of Tea Plant Biology and Utilization, Anhui Agricultural University, 130 Changjiang Ave W., Hefei, Anhui 230036, People's Republic of China; International Joint Laboratory on Tea Chemistry and Health Effects, Anhui Agricultural University, 130 Changjiang Ave W., Hefei, Anhui 230036, People's Republic of China; State Key Laboratory of Tea Plant Biology and Utilization, Anhui Agricultural University, 130 Changjiang Ave W., Hefei, Anhui 230036, People's Republic of China; International Joint Laboratory on Tea Chemistry and Health Effects, Anhui Agricultural University, 130 Changjiang Ave W., Hefei, Anhui 230036, People's Republic of China; State Key Laboratory of Tea Plant Biology and Utilization, Anhui Agricultural University, 130 Changjiang Ave W., Hefei, Anhui 230036, People's Republic of China; International Joint Laboratory on Tea Chemistry and Health Effects, Anhui Agricultural University, 130 Changjiang Ave W., Hefei, Anhui 230036, People's Republic of China; State Key Laboratory of Tea Plant Biology and Utilization, Anhui Agricultural University, 130 Changjiang Ave W., Hefei, Anhui 230036, People's Republic of China; International Joint Laboratory on Tea Chemistry and Health Effects, Anhui Agricultural University, 130 Changjiang Ave W., Hefei, Anhui 230036, People's Republic of China; State Key Laboratory of Tea Plant Biology and Utilization, Anhui Agricultural University, 130 Changjiang Ave W., Hefei, Anhui 230036, People's Republic of China; International Joint Laboratory on Tea Chemistry and Health Effects, Anhui Agricultural University, 130 Changjiang Ave W., Hefei, Anhui 230036, People's Republic of China; State Key Laboratory of Tea Plant Biology and Utilization, Anhui Agricultural University, 130 Changjiang Ave W., Hefei, Anhui 230036, People's Republic of China; International Joint Laboratory on Tea Chemistry and Health Effects, Anhui Agricultural University, 130 Changjiang Ave W., Hefei, Anhui 230036, People's Republic of China; State Key Laboratory of Tea Plant Biology and Utilization, Anhui Agricultural University, 130 Changjiang Ave W., Hefei, Anhui 230036, People's Republic of China; International Joint Laboratory on Tea Chemistry and Health Effects, Anhui Agricultural University, 130 Changjiang Ave W., Hefei, Anhui 230036, People's Republic of China; State Key Laboratory of Tea Plant Biology and Utilization, Anhui Agricultural University, 130 Changjiang Ave W., Hefei, Anhui 230036, People's Republic of China; International Joint Laboratory on Tea Chemistry and Health Effects, Anhui Agricultural University, 130 Changjiang Ave W., Hefei, Anhui 230036, People's Republic of China; State Key Laboratory of Tea Plant Biology and Utilization, Anhui Agricultural University, 130 Changjiang Ave W., Hefei, Anhui 230036, People's Republic of China; International Joint Laboratory on Tea Chemistry and Health Effects, Anhui Agricultural University, 130 Changjiang Ave W., Hefei, Anhui 230036, People's Republic of China; State Key Laboratory of Tea Plant Biology and Utilization, Anhui Agricultural University, 130 Changjiang Ave W., Hefei, Anhui 230036, People's Republic of China; International Joint Laboratory on Tea Chemistry and Health Effects, Anhui Agricultural University, 130 Changjiang Ave W., Hefei, Anhui 230036, People's Republic of China; Biotechnology of Natural Products, Technische Universität München, Liesel-Beckmann-Str. 1, 85354 Freising, Germany; State Key Laboratory of Tea Plant Biology and Utilization, Anhui Agricultural University, 130 Changjiang Ave W., Hefei, Anhui 230036, People's Republic of China; International Joint Laboratory on Tea Chemistry and Health Effects, Anhui Agricultural University, 130 Changjiang Ave W., Hefei, Anhui 230036, People's Republic of China; State Key Laboratory of Tea Plant Biology and Utilization, Anhui Agricultural University, 130 Changjiang Ave W., Hefei, Anhui 230036, People's Republic of China; International Joint Laboratory on Tea Chemistry and Health Effects, Anhui Agricultural University, 130 Changjiang Ave W., Hefei, Anhui 230036, People's Republic of China

## Abstract

Plant uridine diphosphate–dependent glycosyltransferases (UGTs) play a key role in plant growth and metabolism. Here, we examined the evolutionary landscape among UGTs in 28 fully sequenced species from early algae to angiosperms. Our findings revealed a distinctive expansion and contraction of UGTs in the G and H groups in tea (*Camellia sinensis*), respectively. Whole-genome duplication and tandem duplication events jointly drove the massive expansion of UGTs, and the interplay of natural and artificial selection has resulted in marked functional divergence within the G group of the sinensis-type tea population. In Cluster II of group G, differences in substrate selection (e.g. abscisic acid) of the enzymes encoded by UGT genes led to their functional diversification, and these genes influence tolerance to abiotic stresses such as low temperature and drought via different modes of positive and negative regulation, respectively. UGTs in Cluster III of the G group have diverse aroma substrate preferences, which contribute a diverse aroma spectrum of the sinensis-type tea population. All Cluster III genes respond to low-temperature stress, whereas UGTs within Cluster III-1, shaped by artificial selection, are unresponsive to drought. This suggests that artificial selection of tea plants focused on improving quality and cold tolerance as primary targets.

## Teaser

Duplication events have increased the number of uridine diphosphate–dependent glycosyltransferase genes in tea plants, while natural and artificial selection have promoted their functional divergence.

## Introduction

Plants inhabit constantly changing environments, and unfavorable environmental conditions often severely affect plant growth and development. Abiotic stresses such as drought, low temperature, and mineral toxicity can negatively affect the growth, development, and yield of crops and other plants (Verma et al. 2013). Drought and low-temperature stress are some of the major environmental factors affecting the geographical distribution of plants, limiting agricultural production, and threatening food security (Zhu 2016). Tea (*Camellia sinensis*) is one of the world's most important cash crops; it is rich in secondary metabolites and has tremendous medicinal value and cultural importance. Tea plants also provide an ideal perennial model plant for studying the effect of environmental change on terrestrial plants because of their wide distribution, stable ecosystem, stable quality parameters, and long economic life (Han et al. 2018). Recently, some researchers have examined the biological functions of volatile compounds in the response of tea plants to various types of stress and noted that tea plants provide an effective model for studies of the functions of metabolites in plants under multiple types of stress (Jin et al. 2023a, 2023b).

Most cultivated tea plants (*Camellia* L., section *Thea* (L.) Dyer) can be divided into 2 main cultivars: *C. sinensis* var. *assamica* (CSA) and *C. sinensis* var. *sinensis* (CSS). CSS is characterized by small leaves, which are tolerant of cold climates and grow as shrubs or semi-shrubs; CSA has larger leaves, which are more susceptible to cold climates and grow as trees or semi-trees. Therefore, CSA is mainly grown in tropical areas and is typically used to make black tea; CSS is more suitable for cultivation in high-latitude areas and is mainly used for producing high-quality green tea. China currently has a large number of tea plant cultivars, ∼67% of which are CSS; these cultivars play a key role in improving the yield and quality of tea leaves (Kaundun and Matsumoto 2003; Wei et al. 2018; Wu et al. 2022). Analysis of the genomes of different tea cultivars has revealed the mechanisms by which tea plants adapt to environmental changes, including genes related to tea quality and resistance (Zhang et al. 2020; Chen et al. 2023). Lineage-specific gene families in tea plants that have undergone rapid expansions contain many uridine diphosphate (UDP)–dependent glycosyltransferases (UGTs) (Xia et al. 2017). UGTs have been subjected to artificial selection during the breeding of tea plants, and this has resulted in the gain and loss of UGTs in tea plants (Xia et al. 2020). Rapidly expanding gene families involved in disease resistance, secondary metabolism, and growth and development in tea plants also contain UGTs (Wang et al. 2020b). These results suggest that the expansion of UGTs might be closely related to the quality or resistance of tea plants.

Recent studies on the physiological functions of UGTs have further confirmed that UGTs can enhance the stress resistance of tea plants. For example, UGT91Q2 can enhance the cold resistance of tea by glycosylating nerolidol (Zhao et al. 2020), and eugenol can function as a cold and drought signal in tea plants by UGT71A59-mediated glycosylation (Zhao et al. 2022). UGT85A53 can glycosylate (Z)-3-hexenol and thus enhance the defenses of tea plants against *Ectropis obliqua* (Jing et al. 2020), and UGT87E7 can enhance the ability of tea plants to resist fungal infestation by glycosylating SA (Hu et al. 2022). In addition, 4-hydroxy-2,5-dimethylfuran-3(2H)-one (HDMF), a major contributor to the caramel-like aroma of tea, can be specifically catalyzed by UGT74AF3 to generate HDMF glucoside (Chen et al. 2020b). In conclusion, UGTs play an important role in determining the quality of tea and their resistance to biotic and abiotic stress.

UGTs comprise a diverse class of enzymes that are found ubiquitously across various organisms, including plants, animals, fungi, bacteria, and viruses (Bowles et al. 2005). These enzymes play a key role in the transfer of UDP sugar donors to small molecules (sugar acceptors), a process known as glycosylation (Bönisch et al. 2014). Glycosylation facilitates the transport and accumulation of secondary metabolites. In plants, natural pigments, phytohormones, and aroma and flavor compounds often accumulate as glycosides (Song et al. 2018). Most glycosides are bioactive derivatives of secondary metabolites, and previous studies have shown that the substrate specificity and activity of UGT subfamilies vary; this indirectly contributes to the rich and diverse physiological functions of glycosylation in plants (Kurze et al. 2022). However, inferring the expansion of UGTs in tea plants only at the genome level lacks conclusive evidence to explain the relationship of UGT expansion with tea plant quality and stress resistance. Therefore, we analyzed the mechanism underlying the expansion of UGTs in tea at the whole-genome level, and we also analyzed gene duplication events, expression patterns, and the biological functions of UGT members. Overall, our findings provided insights into the evolution and functional diversity of tea UGTs, as well as the mechanism underlying the roles of UGTs in the response of plants to environmental stress.

## Results

### Lineage-specific expansions and contractions of UGTs in tea plants

Phylogenetic analyses of UGTs using a wide range of taxonomic samples from nonmodel systems can provide insights into the evolutionary trajectory among UGTs in plants. We therefore conducted a phylogenetic analysis of 28 representative and fully sequenced plant species, which included taxa ranging from early algae to angiosperms. The results show that UGTs were widely distributed in the plant kingdom and have undergone significant expansions (Supplementary Fig. S1 and Table S1). Our analysis revealed 5 key phylogenetic groups (A, UGT79/91/94/721; D, UGT73; E, UGT71/72/88; G, UGT85; and L, UGT74/75/84) that have undergone significant expansions during the evolutionary history of angiosperms. A total of 297 copies of UGTs in *C. sinensis* were detected, and the above 5 groups had expansion ratios >1 (Supplementary Table S2), which resulted in copy numbers of 47, 49, 37, 30, and 43. Furthermore, the H group (UGT76), which was the second-largest group in the model plant *Arabidopsis*, was present in a single copy in tea plants. This indicates that there was a unique reduction of UGTs in the H group in tea plants. These findings indicate that significant expansions and lineage-specific contractions of UGT members have occurred in tea plants.

**Figure 1.**
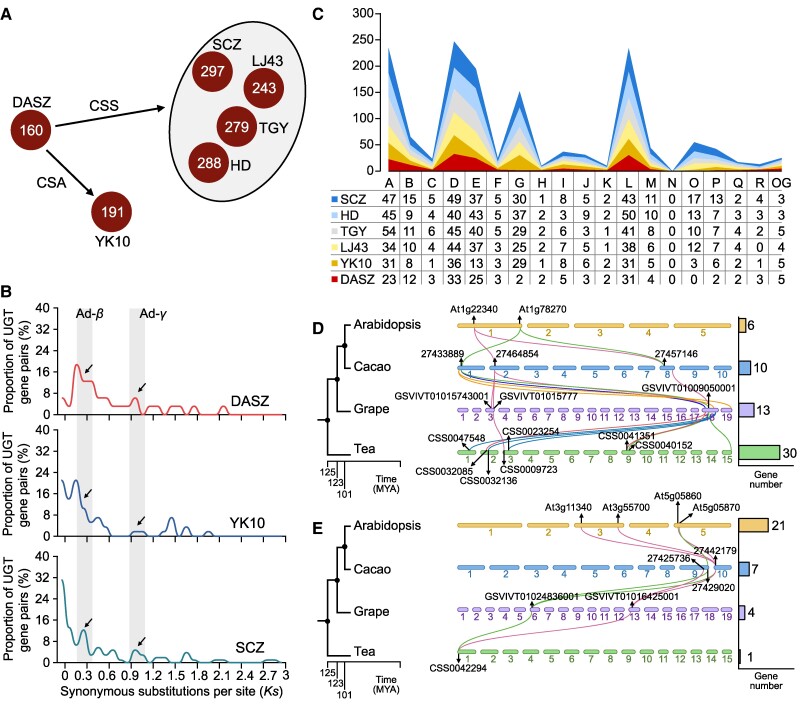
Identification and classification of UGTs in different cultivars of tea plants. **A)** Total UGT identification in different tea plant cultivars. **B)** The synonymous mutation rate (*Ks*) of UGTs' gene pairs in DASZ, YK10, and SCZ, respectively. **C)** Subgroup identification and classification of UGT in different tea plants. The line graph shows the change of UGT group in each breed relative to the total numbers. **D)** Collinearity analysis of G group in Arabidopsis, cacao, grape, and tea (SCZ). **E)** Collinearity analysis of H group in Arabidopsis, cacao, grape, and tea (SCZ).

*Assamica*-type (CSA) and *sinensis*-type (CSS) cultivated tea plants may share a common ancestral origin (Wang et al. 2020b). However, they have evolutionarily diverged, which reflects their marked disparities in growth patterns, stress resistance, and tea quality. To explore variation in the evolution of UGTs among tea cultivars, we studied a range of representative tea cultivars, including the wild tea plant DASZ, CSA-cultivated tea plant yunkang10 (YK10), and CSS-cultivated tea plants including Shuchazao (SCZ), Longjing43 (LJ43), Tieguanyin (TGY), and Huangdan (HD). We observed a notable increase in the total number of UGT genes from wild (DASZ; 160 copies) to cultivated CSA plants (YK10; 191 copies) and to CSS tea plants; the number of UGT genes increased to 297, 243, 279, and 288 copies in SCZ, LJ43, TGY, and HD, respectively (Fig. 1A), indicating that these genes have expanded from wild to cultivated tea plants (CSA and CSS). Given that SCZ has experienced the greatest expansion of UGT gene copies among CSS tea plants, we conducted subsequent studies on this cultivar.

By analyzing the collinearity and gene duplication events of UGTs in tea plants, we obtained 7, 12, and 34 collinearity regions and 28, 45, and 78 tandemly duplicated gene pairs in DASZ, YK10, and SCZ, respectively, suggesting that tandem duplication (TD) events have played an important role in the expansion of UGTs in tea plants. Synonymous mutation rates (*Ks*) for duplicated UGT gene pairs suggested that whole-genome duplication (WGD) events occurred around 25 million years ago (MYA, Ad-*β* event) and ∼90 MYA (Ad-*γ* event, time = *Ks*/2μ, μ = 6.1E−9) (Xia et al. 2017) (Fig. 1B; Supplementary Tables S3 to S5). This indicates that UGT genes have undergone the same genomic duplication events in the 3 tea cultivars, indicating that WGD events have had a major effect on the expansion of UGT genes in tea plants.

We classified UGTs into 18 phylogenetic groups (A–R) and 1 outgroup (OG); the results revealed a significant expansion in the G group from wild to cultivated varieties; only 2 copies were identified in DASZ, but 29 copies were identified in YK10; the average number of copies in CSS populations was 30 (Fig. 1C; Supplementary Table S6). This implies that there might be distinct patterns of expansion of UGTs between CSA- and CSS-cultivated tea plants. Analysis of the collinearity of UGTs among tea plants (SCZ), grape, cocoa, and Arabidopsis revealed that the G group has undergone a more pronounced expansion in tea compared with the other plants, further suggesting that G group members might play a key role in tea plants (Fig. 1D). Although H group members have undergone an expansion in the other species, only 1 to 2 copies of H group members were observed in the different tea cultivars (Fig. 1E), suggesting that there was a lineage-specific contraction of UGTs in the H group.

### H group experiences contraction and involved in tea disease resistance

UGT76, referred to as the H group in this study, comprise the second-largest subfamily of the UGT gene family of Arabidopsis. The H group has been frequently associated with plant immunity and resistance in Arabidopsis. For example, UGT76B1 glycosylates N-hydroxy-pipecolic acid (NHP) or salicylic acid (SA), which plays a role in regulating plant basal immunity and systemic acquired resistance (SAR) (Bauer et al. 2021; Mohnike et al. 2021). However, H group has experienced a significant contraction in tea plant (SCZ) and retained only a single copy (*CSS0042294*).

**Figure 2.**
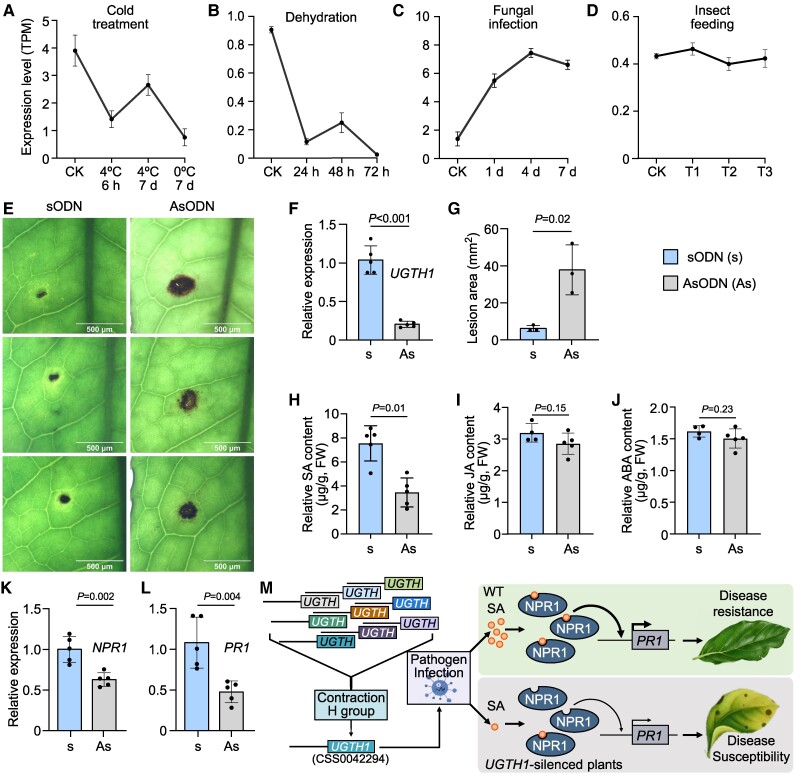
H group retains significant disease resistance. **A** to **D)** Expression level of *UGTH1* in low temperature, dehydration, and fungal infection and *E. oblique* feeding on tea plants, respectively. CK, control; 4℃_6 h, 4 ℃ for 6 h; 4℃_7d, 4 ℃ for 7 days; 0℃_7d, 0 ℃ for 7 days; 24h, 48 h, 72 h, tea samples were treated with PEG-6000 for 24, 48, and 72 h, respectively; 1d, 4d, 7d, fungi infected tea samples for 1, 4, and 7 days, respectively. T1 to T3, biological replication of the *E. oblique* feeding on tea leaves. **E)** Disease symptoms after fungal infection for 4 days were observed under a stereomicroscope after suppressing *UGTH1*. **F)** The relative expression of control (sODN) and *UGTH1*-suppressed (AsODN) in tea plants. **G)** The lesion area of control (sODN) and *UGTH1*-suppressed (AsODN) in tea plants after fungal infection for 4 days. **H** to **J)** The relative contents of hormones of control (sODN) and *UGTH1*-suppressed (AsODN) in tea plants before fungal infection. FW, fresh weight. **K** to **L)** Relative expression levels of *NPR1* and *PR1* in tea plants of control (sODN) and *UGTH1*-suppressed (AsODN). **M)** Working models of UGTH1 from H group in response to disease resistance in tea plants. Data were expressed as the mean ± Sd from at least 5 biological replicates. All statistical analysis was performed by Student's *t*-tests.

To determine the biological roles of H group members in tea plants, we analyzed the transcript levels of *CSS0042294* (referred to as UGTH1 in this study) in tea plants under various types of stress, including low temperature, drought, pathogen infection, and herbivory by *E. obliqua* (Fig. 2, A to D). The expression level of *UGTH1* increased after tea plants were infested with pathogens, indicating that *UGTH1* can be strongly induced by disease stress. Antisense oligonucleotides (AsODNs) are nucleic acid fragments that specifically bind to the DNA or mRNA of a target gene, thereby inhibiting the expression of that gene (Crooke et al. 2021). AsODNs have been widely used for the transient suppression of genes in tea plants (Jing et al. 2019; Zhao et al. 2020; Jin et al. 2023a; Lu et al. 2023). To clarify whether *UGTH1* is involved in the response to disease stress in tea plants, we transiently suppressed *UGTH1* using AsODN and infected *UGTH1*-suppressed tea plants with a fungal pathogen. The results revealed a significant increase in the lesion area of tea plants after the suppression of *UGTH1* compared with the control (sODN), which indicated that the suppression of *UGTH1* reduces the disease resistance of tea plants (Fig. 2, E to G). Phytohormones play an important role in the defense of plants against disease stress (Zhou and Zhang 2020; Zhao and Li 2021; Hu et al. 2022), and we found that they can effectively improve the disease resistance of tea plants (Supplementary Fig. S2). In this study, the relative content of SA in tea plants was significantly reduced after suppressing *UGTH1* (Fig. 2, H to J), and the expression levels of the disease resistance genes *NPR1* and *PR1* were also reduced (Fig. 2, K and L), which decreased the disease resistance of tea plants. These results suggested that UGTH1 could be involved in tea plant disease resistance by indirectly affecting the content of SA. However, we did not find in vitro evidence suggesting that UGTH1 can glycosylate NHP or SA (Supplementary Fig. S3). Therefore, these results led us to hypothesize that H groups in tea plants may be involved in tea plant disease resistance by glycosylating certain compounds to affect SA content (Fig. 2M), and their physiological functions need to be further investigated.

### Natural and artificial selection drives the expansion of G group members in *sinensis*-type tea plants

**Figure 3.**
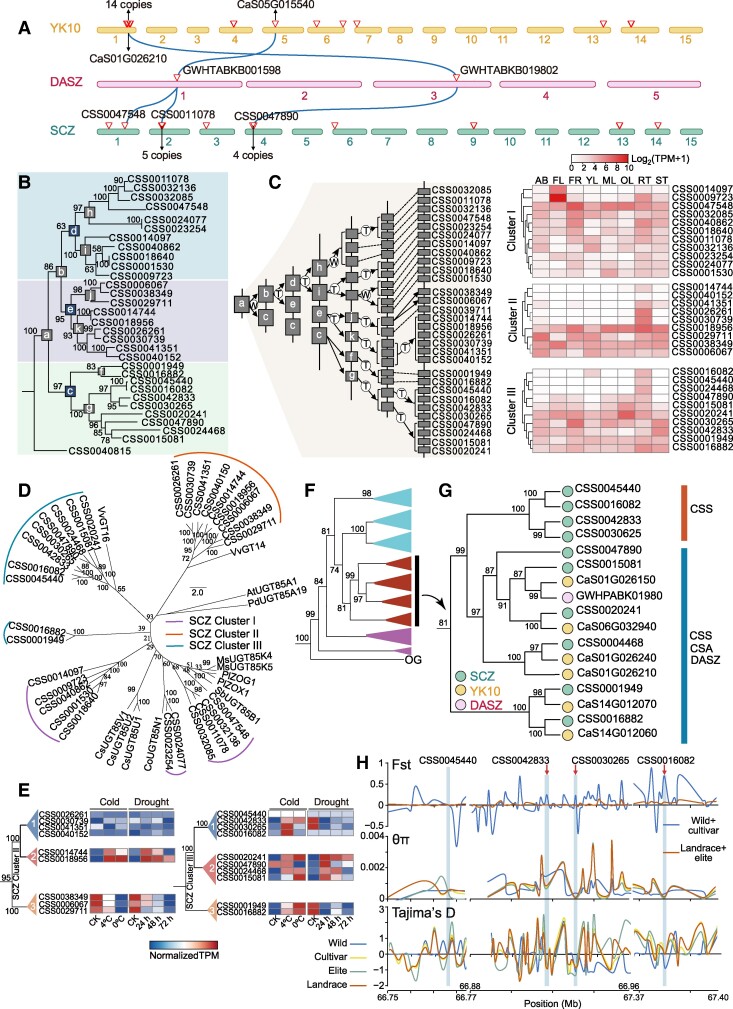
Evolutionary analysis of the G group in tea plants. **A)** Collinearity analysis of G group UGTs in DASZ, YK10, and SCZ. **B)** Phylogenetic relationships of the UGT genes for G group in the SCZ. CSS0040815 from the OG group in the homologous classification was selected as an outgroup. **C)** Hypothetical evolutionary histories of the UGTs genes in G group (left). The letters T and W in the schematic diagram showing the hypothetical origins of UGT genes indicate tandem duplication and WGD, respectively. The heat map (right) represents the transcript levels of G group genes in different tissues of tea plant, the heat map color is calculated using log_2_(TPM + 1). AB, apical bud; FL, flower; FR, fruit; YL, young leaf; ML, mature leaf; OL, old leaf; RT, root; ST, stem. **D)** Phylogenetic relationships of G group (UGT85) genes in plants. Phylogenetic relationships were reconstructed using IQ-Tree (Ultrafast bootstrap = 5000), and sequences were aligned using MAFFT. AtUGT85A1 (AAF18537), *Arabidopsis thaliana*; PdUGT85A19 (ABV68925), *Prunus dulcis*; SbUGT85B1 (AAF17077), *Sorghum bicolor*; MsUGT85K4 (AEO45781), MsUGT85K5 (AEO45782), *Manihot esculenta*; CoUGT85N1 (AEB61489), *Consolida orientalis*; VvGT14 (XP_002285770.1), VvGT16 (XP_002263158.1), *Vitis vinifera*; CsUGT85V1 (KF446241), CsUGT85U1 (KF446243), CsUGT85U2 (KF446242), *Crocus sativus*; PlZOG1 (AAD04166), PlZOX1 (AAD51778), *Phaseolus lunatus*. UGT85A1 was selected as an outgroup. **E)** Expression patterns of Cluster II (left) and Cluster III (right) UGTs from G group under abiotic stresses. **F** to **G)** Phylogenetic relationships of G group genes in DASZ, YK10, and SCZ. Phylogenetic tree was reconstructed using IQ-Tree (Ultrafast bootstrap = 5000), and sequences were aligned using MAFFT. CSS0040815, CSS0032998, and CSS0033747 from the OG group in the homologous classification was selected as an outgroup. **H)** Selective sweep regions around Cluster III-1 genes' locus were evaluated by different summary statistics. The blue bar denotes the location of the genes within Cluster III-1. The arrow denotes the genes subjected to selection. The statistics were calculated separately for populations from wild, cultivator, landrace, and elite accessions.

UGT85 members, denoted as the G group in this study, typically have a preference for glycosylating monoterpenes, diterpenes, and apocarotenoids (Kurze et al. 2022). In our study, we observed a significant expansion of G group members within tea plants. This expansion suggests that there is a potential link between the expansion of the G group and tea quality or resistance. Analysis of the collinearity of G group members in wild tea plants (DASZ), CSA (YK10), and CSS (SCZ) revealed that the genes in YK10 and SCZ were homologous to G group genes in DASZ (Fig. 3A). Notably, *GWHTABKB001598*, which originated from DASZ, diverged into *CSS0047548* and *CSS0011078* in SCZ. This pattern of divergence contrasts with that observed in YK10. This led us to hypothesize that the expansion of G group members in CSS might have distinct characteristics compared with DASZ and YK10.

We set up branch nodes (a–k) in the phylogenetic tree of the G group of SCZ cultivars and compared the protein sequences of each branch to obtain consistent sequences for each branch. Next, the most parsimonious evolutionary history of the G group in SCZ was inferred by calculating the *Ks* values of these consistent sequences (Fig. 3B). The results showed that after a round of WGD and TD events, SCZ acquired 3 ancestral genes (labeled as d, e, and c, respectively), and this group of genes continued to expand in the SCZ under the combined effect of WGD and TD events, suggesting that WGD and TD jointly drove the significant expansion of the G group in SCZ (Fig. 3C; Supplementary Fig. S4). In addition, UGTs from different ancestral genes (d, e, and c) formed different clusters in the phylogenetic tree, including Cluster I, Cluster II, and Cluster III, and spatial patterns of the expression of genes in Cluster I were consistent across all tissues; Clusters II and III were distinctly clustered. Some genes in Clusters II and III were expressed in all tissues, whereas genes in other clusters were exclusively expressed in the roots (Fig. 3C; Supplementary Table S7).

Phylogenetic analysis of UGTs from other species revealed differentiation among members of Cluster II in tea plants (ultrafast bootstrap support value ≥ 95), in light of the opposing responses of UGTs from Cluster II-2 and 3 to abiotic stresses such as low temperature and drought; we hypothesized that genes in Cluster II have functionally diverged (Fig. 3, D and E; Supplementary Table S8). The UGTs in Cluster III all responded to low temperature. However, only the UGTs in Cluster III-2 responded to drought stress, and the expression of UGTs in Cluster III-1 was not significantly altered under drought stress. Therefore, we hypothesized that UGTs in Cluster III have also functionally diverged (Fig. 3E; Supplementary Table S8).

We performed phylogenetic analyses of UGTs in the G group in DASZ (wild tea), YK10 (CSA), and SCZ (CSS). A maximum likelihood (ML) tree was constructed using IQ-TREE and 5,000 ultrafast bootstrap replicates. The resulting phylogenetic tree had some internodes with ultrafast bootstrap support values ≤ 95, which were considered unreliable (Minh et al. 2013). According to the reliable support values we obtained at these branches, we found that the Cluster III-1 branch was exclusive to SCZ (with ultrafast bootstrap support values = 99), whereas other genes might have evolved in parallel in DASZ and YK10 (Fig. 3, F and G; Supplementary Fig. S5). This led us to ask whether ClusterIII-1 might represent a group of genes specific to cultivated tea plants. To assess this, we scanned for genomic selective sweep regions across wild, cultivator, landrace, and elite populations (Xia et al. 2020); we then evaluated them using ratios of nucleotide diversity (θπ), genetic distance (*F*_ST_), and Tajima's *D*. We found that UGTs from ClusterIII-1 (*CSS0016082*, *CSS0042833*, and *CSS0030265*) had reduced nucleotide diversity, showed significant differences in *F*_ST_, and Tajima's D << 0 compared with wild populations, suggesting that these genes might have been subjected to artificial selection (Fig. 3H).

### Divergent substrate selectivity of the enzymes encoded by duplicated gene pairs of *UGT85A53* and *CSS0029711* in Cluster II of G group leads to distinct roles in cold and drought response

**Figure 4.**
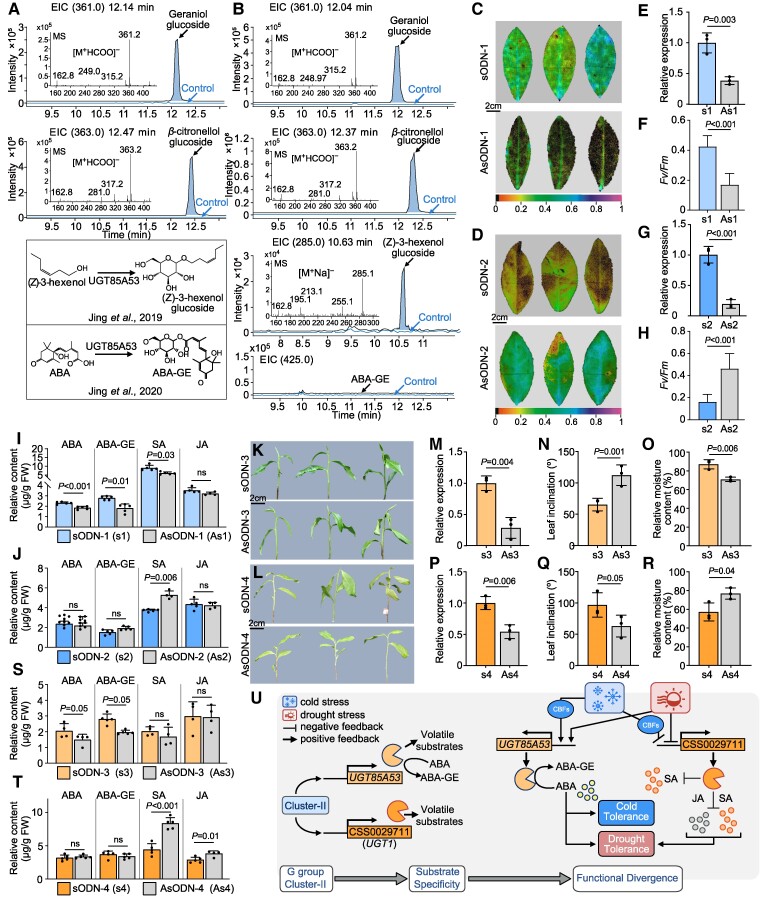
Functional validation of UGT genes in Cluster II from G group. **A)** LC–MS/MS analyses of enzymatic products formed by UGT85A53. The mass spectra of the enzymatic reaction products produced by recombinant UGT85A53 are listed. **B)** LC–MS/MS analyses of enzymatic products formed by UGT1. The mass spectra of the enzymatic reaction products produced by recombinant UGT1 are listed. **C)** Chlorophyll fluorescence images of control (sODN-1) and *UGT85A53*-suppressed (AsODN-1) tea plants under 4 ℃ for 48 h. The color-coded values indicate the degree of damage, with smaller values indicating more severe damage. **D)** Chlorophyll fluorescence images of control (sODN-2) and *UGT1*-suppressed (AsODN-2) tea plants under 4 ℃ for 48 h. **E, G)** Relative expression of control (sODN-1/sODN-2) and *UGT85A53*-suppressed (AsODN-1)/*UGT1*-suppressed (AsODN-2) in tea plants, respectively. **F, H)** Statistical analysis of maximum photochemical efficiency of photosystem II (*F*_v_/*F*_m_*)* in **E)** and **G)**. **I, J)** Relative content of hormones in control (sODN-1/sODN-2) and *UGT85A53*-suppressed (AsODN-1)/*UGT1*-suppressed (AsODN-2) in tea plants under 4 ℃, respectively. **K, L)** The tea plant phenotypes of control (sODN-3/sODN-4) and *UGT85A53*-suppressed (AsODN-3)/*UGT1*-suppressed (AsODN-3) after drought stress for 24 h, respectively. **M, P)** Relative expression of control (sODN-3/sODN-4) and *UGT85A53*-suppressed (AsODN-3)/*UGT1*-suppressed (AsODN-4) in tea plants, respectively. **N, Q)** Leaf inclination of tea plants in control (sODN-3/sODN-4) and *UGT85A53*-suppressed (AsODN-3)/*UGT1*-suppressed (AsODN-4) after drought stress for 24 h, respectively. **O, R)** Relative moisture content of tea plants in control (sODN-3/sODN-4) and *UGT85A53*-suppressed (AsODN-3)/*UGT1*-suppressed (AsODN-4) after drought stress for 24 h, respectively. **S, T)** Relative content of hormones in control (sODN-3/sODN-4) and *UGT85A53*-suppressed (AsODN-3)/*UGT1*-suppressed (AsODN-4) in tea plants after drought stress for 24 h, respectively. **U)** Working models of UGT85A53 and UGT1 from Cluster II in response to cold and drought stress response in tea plants. Images in **C), D), K)**, and **L)** were digitally extracted for comparison. Data were expressed as the mean ± Sd from at least 3 biological replicates. All statistical analysis was performed by Student's *t*-tests.

UGTs in Cluster II of the G group might be functionally diverged. To evaluate this hypothesis, we selected *CSS0018956* and *CSS0029711* (referred to as UGT85A53 and UGT1 in this study, respectively) as representatives of Clusters II-2 and II-3, respectively, which exhibit opposite responses to abiotic stresses. Among them, UGT85A53 has been shown to glycosylate *cis*-3-hexenol and abscisic acid (ABA) (Jing et al. 2019, 2020). The enzyme activity results showed that UGT1 had the highest catalytic activity for geraniol and *β*-citronellol, and the free UDP content generated was 650.2 *μ*m and 584.8 *μ*m, respectively. UGT1 catalyzed *cis*-3-hexenol and ABA, the optimal substrates for UGT85A53, and the free UDP content generated was 179.9 *μ*m and 76.19 *μ*m, respectively, which was slightly higher than that observed in the control (58.0 *μ*m) (Supplementary Fig. S6A). To compare differences in substrate preferences between duplicated gene pairs, we determined the quantity of products of the above substrates, which served as an indicator of catalytic activity, using liquid chromatography (LC)–MS/MS. Our findings indicated that both UGT85A53 (Fig. 4A) and UGT1 (Fig. 4B) were able to glycosylate geraniol, *β*-citronellol, and *cis*-3-hexenol to produce the corresponding glycoside products. However, unlike UGT85A53, which can glycosylate ABA to generate ABA-glucose ester (ABA-GE), UGT1 did not glycosylate ABA or other hormones in assays. These findings indicate that the substrate selectivity of duplicated gene pairs in Cluster II of G group was distinct, a consistent preference for volatile compounds was observed, but preferences for nonvolatile compounds (especially hormones) were variable.

Substrate selectivity might indirectly contribute to functional divergence in Cluster II of the G group. To assess these hypotheses, we transiently suppressed *UGT85A53* and *UGT1* in tea plants using AsODN, which selectively reduces the transcript levels of target genes without reducing the expression levels of other genes (Supplementary Fig. S7). Subsequently, suppressed tea plants were subjected to low-temperature and drought treatments. The results showed that suppressing *CSS0018956* (AsODN-1) at 4 °C significantly reduced the maximum photochemical efficiency of photosystem II (*F*_v_/*F*_m_) compared with the control group (sODN-1), which reflects increased damage to tea plants (Fig. 4, C, E. and F). Conversely, suppressing *UGT1* (AsODN-2) resulted in less damage at 4 °C and significantly higher *F*_v_/*F*_m_ values in tea plants, indicating that the cold stress resistance of tea plants was improved (Fig. 4, D, G, and H). *CBF*s (C-repeat binding factors) are some of the most important and representative regulatory pathways in the plant cold-signaling pathway, and *CBF*s bind to and activate the expression of *cis*-elements in the promoters of cold-responsive genes or UGTs, which are involved in the cold resistance of tea plants (Hu et al. 2020; Zhao et al. 2020, 2022). Thus, we investigated the effects of transiently suppressing *CsCBFs* on gene expression levels within Cluster II (Supplementary Fig. S8 and Fig. S9). Our findings showed that suppressing *CsCBF4* and *CsCBF5* led to the reduced expression of *UGT85A53* (Supplementary Fig. S10) and inhibiting *CsCBF3*, *CsCBF4*, and *CsCBF6* reduced the expression of *UGT1* (Supplementary Fig. S11). These findings suggest that Cluster II genes might rely on different *CBF* pathways to enhance tea cold tolerance. Under drought treatment, inhibiting *UGT85A53* (AsODN-3) promoted wilting and a notable increase in the leaf inclination angle compared with the control (sODN-3), indicating that the drought tolerance of tea plants was reduced (Fig. 4, K and M to O). In contrast, suppressing *UGT1* (AsODN-4) led to reduced wilting and a significant decline in the leaf inclination angle compared with the control (sODN-4), indicating that the drought resistance of tea plants was enhanced (Fig. 4, L and P to R).

Phytohormones play an important role in regulating the response of plants to low-temperature and drought stress (Miura and Ohta 2010; Hu et al. 2013; Nakashima et al. 2014). We also found that phytohormones are effective in improving the cold and drought resistance of tea plants (Supplementary Fig. S12); their changes affect the stress resistance of tea plants and can therefore be used as indicators of the stress resistance of tea plants (Han et al. 2018). In this study, suppressing *UGT85A53* reduced the levels of ABA and ABA-GE under both low-temperature and drought stress (Fig. 4, I and S), suggesting that UGT85A53 regulates the low-temperature and drought resistance of tea plants by glycosylating ABA. Conversely, levels of SA increased after suppressing *UGT1* under low-temperature stress (Fig. 4J), and both the SA and JA content increased under drought (Fig. 4T). These findings suggest that the duplicated gene pairs in Cluster II of the G group can play a role in defense against abiotic stresses, such as low-temperature and drought stress, through different regulatory mechanisms (Fig. 4U). This diversification in defense mechanisms highlights the adaptability of tea plants to environmental stress.

### Duplicate domestication genes *CSS0030265* and *CSS0020241* in Cluster III of G group exhibit conserved cold but divergent drought tolerance

**Figure 5.**
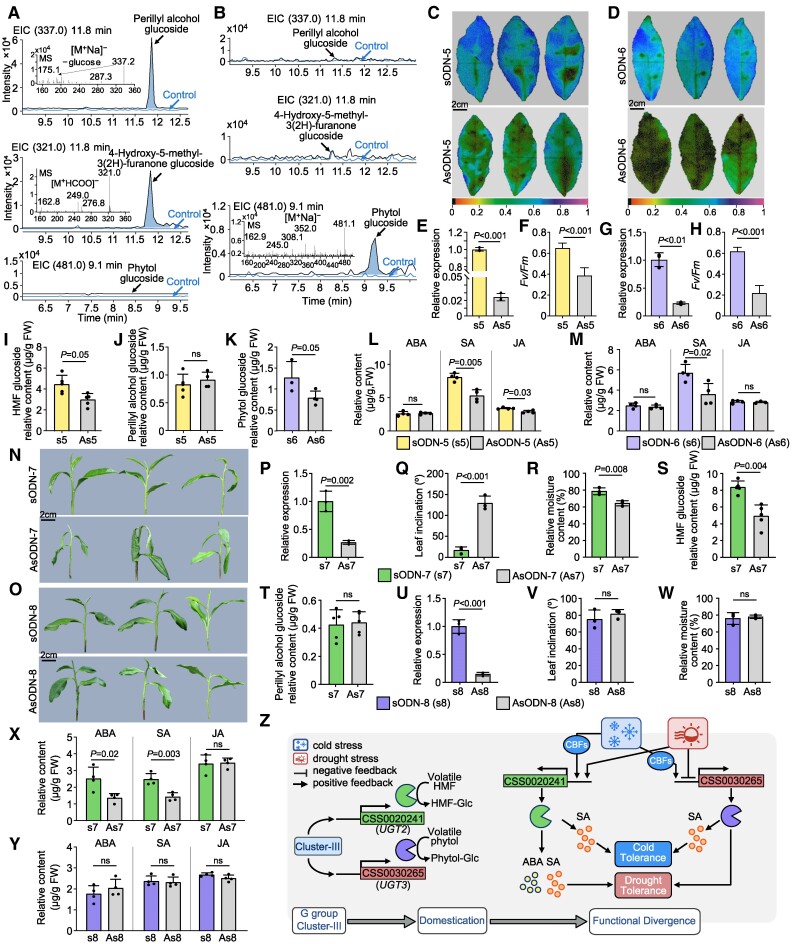
Functional validation of UGT genes in Cluster III from G group. **A)** LC–MS/MS analyses of enzymatic products formed by UGT2. The mass spectra of the enzymatic reaction products produced by recombinant UGT2 are listed. **B)** LC–MS/MS analyses of enzymatic products formed by UGT3. The mass spectra of the enzymatic reaction products produced by recombinant UGT3 are listed. **C)** Chlorophyll fluorescence images of control (sODN-5) and *UGT2*-suppressed (AsODN-5) in tea plants under 4 ℃ for 48 h. The color-coded values indicate the degree of damage, with smaller values indicating more severe damage. **D)** Chlorophyll fluorescence images of control (sODN-6) and *UGT3*-suppressed (AsODN-6) in tea plants under 4 ℃ for 48 h. **E, G)** Relative expression of control (sODN-5/sODN-6) and *UGT2*-suppressed (AsODN-5)/*UGT3*-suppressed (AsODN-6) in tea plants, respectively. **F, H)** Statistical analysis of maximum photochemical efficiency of photosystem II (*F*_v_/*F*_m_) in **C), D)**. **I**, and **J)** The relative content of HMF-glucoside/perillyl alcohol-glucoside of control (sODN-5) and *UGT2*-suppressed (AsODN-5) in tea plants, respectively. **K)** The relative content of phytol-glucoside of control (sODN-6) and *UGT3*-suppressed (AsODN-6) in tea plants. **L, M)** Relative content of hormones in control (sODN-5/sODN-6) and *UGT2*-suppressed (AsODN-5)/*UGT3*-suppressed (AsODN-6) in tea plants under 4 ℃, respectively. **N, O)** The tea plant phenotypes of control (sODN-7/sODN-8) and *UGT2*-suppressed (AsODN-7)/*UGT3*-suppressed (AsODN-8) after drought stress for 24 h, respectively. **P, U)** Relative expression of control (sODN-7/sODN-8) and *UGT2*-suppressed (AsODN-7)/*UGT3*-suppressed (AsODN-8) in tea plants, respectively. **Q, V)** Leaf inclination of tea plants in control (sODN-7/sODN-8) and *UGT2*-suppressed (AsODN-7)/*UGT3*-suppressed (AsODN-8) after drought stress for 24 h, respectively. **R, W)** Relative moisture content of tea plants in control (sODN-7/sODN-8) and *UGT2*-suppressed (AsODN-7)/*UGT3*-suppressed (AsODN-8) after drought stress for 24 h, respectively. **S, T)** The relative content of HMF-glucoside/perillyl alcohol-glucoside of control (sODN-7) and *UGT2*-suppressed (AsODN-7) in tea plants, respectively. **X, Y)** Relative content of hormones in control (sODN-7/sODN-8) and *UGT2*-suppressed (AsODN-7)/*UGT3*-suppressed (AsODN-8) in tea plants after drought stress for 24 h, respectively. **Z)** Working models of *UGT2* and *UGT3* from Cluster III in response to cold and drought stress response in tea plants. Images in **C)**, **D)**, **N)**, and **O)** were digitally extracted for comparison. Data were expressed as the mean ± Sd from at least 3 biological replicates. All statistical analysis was performed by Student's *t*-tests.

Duplicate domestication genes in Cluster III might also be functionally differentiated. To investigate the functional divergence among Cluster III members, we examined 2 genes, *CSS0030265* (referred to as UGT3 in this study) and *CSS0020241* (referred to as UGT2 in this study), which served as representatives of Clusters III-1 and III-2, respectively. The results of the enzyme activity assays of the recombinant proteins UGT2 and UGT3 showed that UGT2 had the highest catalytic activity for perillyl alcohol and 4-hydroxy-5-methyl-3(2H)-furanone (HMF), and the free UDP content generated for these 2 substrates was 545.9 *μ*m and 475.2 *μ*m, respectively (Supplementary Fig. S6C). UGT2 was able to glycosylate perillyl alcohol and HMF to produce the corresponding O-glucoside in vitro (Fig. 5A). By contrast, UGT3 had the highest catalytic activity for phytol and generated 597.9 *μ*m free UDP (Supplementary Fig. S6B); it was able to glycosylate phytol to produce phytol-O-glucoside in vitro (Fig. 5B). This suggests that the duplicated gene pairs of Cluster III under artificial selection tended to differ in substrate selection, which indirectly contributes to the diversity of tea plant aroma components.

The response of UGTs in Cluster III to low-temperature and drought stress suggests that they might be functionally diverged. To examine this hypothesis, we conducted low-temperature and drought treatments after transiently suppressing *UGT2* and *UGT3* in tea plants. After transiently suppressing *UGT2* (AsODN-5) and *UGT3* (AsODN-6) at 4 °C, the degree of damage was significantly higher in suppressed tea plants than in control plants (sODN-5,6, Fig. 5, C to E and G), and the *F*_v_/*F*_m_ was significantly lower in suppressed plants than in control plants (Fig. 5, F and H), indicating that the cold tolerance of tea plants was reduced in suppressed plants. In addition, the relative content of HMF-O-glycosides was decreased in tea plants after the transient suppression of *UGT2*; no significant change was observed in the content of perillyl alcohol-O-glycosides, suggesting that UGT2 mainly mediated the synthesis of HMF-O-glycosides in tea plants (Fig. 5, I and J); the relative content of phytol-O-glycosides in tea plants also decreased after the transient suppression of *UGT3* (Fig. 5K), suggesting that UGT3 could mediate the synthesis of phytol-O-glycosides in tea plants. The expression of *UGT2* decreased when *CsCBF4* (Supplementary Fig. S13) was suppressed, and the expression of *UGT3* decreased when *CsCBF2*, *CsCBF3*, and *CsCBF6* (Supplementary Fig. S14) were suppressed, suggesting that UGT2 and UGT3 relied on distinct *CBF* pathways to enhance the cold tolerance of tea plants.

Under drought conditions, the degree of wilting and leaf inclination angle of tea plants significantly increased, and the relative water content was significantly reduced after *UGT2* was transiently suppressed (Fig. 5, N and P to R), the relative content of HMF-O-glycosides decreased (Fig. 5S), while the relative content of perillyl alcohol-O-glycosides did not change significantly (Fig. 5T), indicating that the transient suppression of *UGT2* reduced the drought tolerance of tea plants. By contrast, there was no significant difference in the degree of wilting, leaf inclination angle, and water content of the tea plants after *UGT3* was transiently suppressed (Fig. 5, O and U to W), suggesting that UGT3 did not significantly affect the drought tolerance of tea plants. In addition, the transient suppression of *UGT2* and *UGT3* in tea plants indirectly affected the significant reduction in the relative content of SA in tea plants under low-temperature conditions (Fig. 5, L and M), which led to a reduction in the cold tolerance of tea plants. Under drought conditions, the content of SA and ABA in tea plants was significantly reduced after the transient suppression of *UGT2* (Fig. 5X), and the relative content of ABA did not change significantly after the transient suppression of *UGT3* (Fig. 5Y), which might explain why the suppression of *UGT3* did not have a significant effect on the drought tolerance of tea plants. These findings suggest that genes within Cluster III are functionally diverged, and this could contribute to their variable responses to abiotic stress (Fig. 5Z). Notably, genes within Cluster III-1, which have been mostly subjected to artificial selection, did not significantly contribute to responses to drought stress in tea. This observation implies that cold tolerance might have been selectively favored in the CSS population, which was likely driven by the prevailing climatic conditions.

## Discussion

In this study, we examined the glycosyltransferase 1 (GT1) family responsible for the glycosylation of plant secondary metabolites. We identified a total of 28 plant UGTs, which indicated that UGT family members have undergone a significant expansion in terrestrial plants. The results of our study revealed a significant expansion and lineage-specific contraction of UGTs in tea plants, which might be related to the environmental adaptation of tea plants.

Tea plants are rich in secondary metabolites, and the flavor substances in tea are key quality indicators. Many volatile aroma substances are stored in tea plants as glycosidically bound volatiles (GBVs), which can release volatile aroma compounds after hydrolysis and participate in the formation of tea aroma (Nishikitani et al. 1996; Ananthakumar et al. 2006; Guo et al. 1996). Recent population sequencing of tea plants has revealed that tea plants have retained gene families related to tea quality and resistance for long evolutionary periods, including a large number of UGTs, and UGTs have undergone a rapid expansion in tea plants (Xia et al. 2020). In addition, genes associated with flavor metabolism and cold tolerance have been subjected to stronger selection in the CSS population than in the CSA population (Wang et al. 2020b). Therefore, the expansion of UGTs in tea plants not only contributes to the metabolism of flavor substances but also enhances the resistance of tea plants to various biotic and abiotic stresses (Song et al. 2018). In this study, we examined the G group of UGTs, which has undergone a significant expansion and is the most representative UGT subfamily, to test conjectures derived from above genome-level studies of tea plants.

The first major finding of our study relates to the functional divergence of UGTs in tea plants. A previous study has shown that *Camellia* plants originated around 14.30 MYA, and they diversified at ∼6.67 MYA (Wu et al. 2022). In this study, we selected a range of representative *Camellia* plants that diverged from a common ancestor <5 MYA. Our study revealed significant differences in the copy number of UGTs and their functions among tea plants with close genetic backgrounds over a short divergence time, and the copy number of UGTs was greater in cultivated tea (CSA and CSS) than in wild tea (DASZ). In the G group, for example, the gene copy number increased from 2 in DASZ (wild tea) to 29 copies in YK10 (CSA) and 30 copies in SCZ (CSS), and the expansion of UGTs in the G group with the same origin in SCZ differed from that in YK10. Further analysis of the G group in SCZ cultivars revealed that WGD events and TD events together promoted the expansion of the G group via natural selection, and significant functional divergence was observed among these duplicated gene pairs. For example, the enzymes encoded by the duplicated genes in Cluster II in group G (UGT85A3 and UGT1) show convergent substrate preferences for volatile compounds (e.g. aroma substances) and divergent substrate preferences for nonvolatile compounds (e.g. ABA). Differences in preferences for ABA have led to divergence in their functions; in other words, UGT85A53 and UGT1 might be involved in the defense of tea plants against abiotic stresses such as low-temperature and drought through positive and negative regulatory pathways, respectively. The results revealed that natural selection has shaped the evolutionary trajectory of tea plants by affecting the quality of tea and promoting differentiation in the stress resistance of tea plants, which reflects the diversification of tea plant defense strategies during the evolutionary process. Although we only examined a representative subfamily of UGTs in our study, the results suggest that more studies of UGTs could shed light on the complex relationships between the mechanism underlying the expansion of UGTs and the quality and stress resistance of tea plants.

Our study also provides evidence for the perception that aroma quality and cold tolerance are the main targets of artificial selection in tea plants. Tea plants in the Yangtze River Basin and eastern China are susceptible to low temperatures in the early spring and winter, and most CSA cultivars are unable to survive in these areas. The accumulation of secondary metabolites associated with tea quality (e.g. catechins, caffeine, theanine, and terpene volatiles) and gene families (e.g. UGTs) in cultivated tea plants might be a result of both artificial and natural selection (Xia et al. 2020). As tea plants began to be subjected to artificial selection, the CSS and CSA populations diverged during the domestication process, CSS experienced stronger selection for tea flavor and cold tolerance than CSA during the domestication process (Wang et al. 2020b). Based on phylogenetic analyses of DASZ, YK10, and SCZ, we found that Cluster III-1 of the G group comprised a unique branch in SCZ, and most of the genes in this branch show strong domestication signals. G group members generally have a preference for glycosylating volatile compounds (Kurze et al. 2022). In this study, we found that the duplicated gene pairs of Cluster III in the G group, UGT2 and UGT3, glycosylated different aroma substrates, suggesting that the UGTs in Cluster III can bind to a more diverse set of aroma substrates, which might contribute to the diversification of flavor substances in the CSS population. Both UGT3 (Cluster III-1) and UGT2 (Cluster III-2) in the G group positively regulated cold tolerance in tea plants; however, the genes that were artificially domesticated in Cluster III-1 were not significantly altered under drought stress. For example, UGT3 had no significant effect on drought stress in tea plants, which provides direct evidence that cold tolerance was the main target of the domestication process of CSS populations. The present results indicate that the significant expansion and functional differentiation of the G group during the domestication of the CSS population of tea plants not only promoted the diversification of flavor components but also enhanced the adaptation of tea plants to abiotic stresses, which likely plays an important role in enhancing cold resistance. One major limitation of this study is that we did not examine the functions of UGTs in CSA populations, but as the genomes of more CSA populations are sequenced, in-depth analyses of the functional differences of UGTs between CSS and CSA populations will be the major focus of our follow-up work. These studies will enhance our understanding of the evolutionary history of cultivated tea plants.

We also examined the physiological functions of genes in the H group (UGT76) in tea plants. UGT76B1 can play a role in plant immunity by glycosylating NHP and SA in Arabidopsis (Bauer et al. 2021; Mohnike et al. 2021). However, UGTH1, the only copy of H group members retained in tea plants, does not glycosylate NHP and SA. The expression of UGTH1 was upregulated in tea plants after fungal infestation, suggesting that this gene retains its physiological activity related to disease resistance in tea plants. The transient suppression of *UGTH1* was followed by a reduction in the SA content in tea plants, along with a decrease in disease resistance, suggesting that UGTH1 might regulate the disease resistance of tea plants by indirectly affecting the level of SA. The lack of SA glycosylation by UGTH1 is compensated for by UGT87E7 from the J group, which can glycosylate SA to form SA glucose ester (SGE) and positively regulate disease resistance in tea plants (Hu et al. 2022), while UGT95B17 from the Q group has been reported to glycosylate 2,4-dihydroxybenzoic acid (2,4-DHBA), which is the hydroxylated product of SA; it thus improves disease resistance in tea plants (Lu et al. 2023). These findings imply that the H group might be involved in the disease resistance of tea plants by affecting the SA content, which is altered via the glycosylation of certain substrates; this maintains the functional diversity and homeostasis of UGTs in tea plants. These findings provide valuable information that could aid future studies of the physiological mechanisms of UGTH1 in tea disease resistance.

In conclusion, our data suggest that the interaction of natural and artificial selection promotes the functional diversity of UGTs in tea plants, and the UGTs retained in this process can fulfill both quality and resistance requirements of tea plant to adapt to environmental changes. Our findings regarding the role of UGTs in the adaptive evolution and quality formation of tea plants enhance our understanding of the evolution and functional diversity of UGT gene family members and provide valuable insights into the diversity of defense strategies as well as how selection has shaped substrate preferences of UGTs in tea plants.

## Materials and methods

### Plant material and growth conditions

One-year-old healthy tea seedlings (*C. sinensis* (L.) O. Kuntze var. *sinensis*, ‘Shuchazao’) were obtained from Dechang Seedling Company (Luan, Anhui, China). Tea seedlings were obtained in March 2023 for fungal infestation, and tea seedlings obtained in July 2023 were used for drought and low-temperature treatment. All tea seedlings had the same height and growth status and were free of pests and diseases. All treatments and sample harvesting were carried out on the 1st and 2nd leaves of tea seedlings unless otherwise noted, and leaves harvested from 2 tea seedlings comprised a single biological replicate.

### Identification of UGT gene family members in tea plants

Genome sequences of tea plants were downloaded from TPIA (http://tpia.teaplants.cn/) (Gao et al. 2023), including the wild tea plant DASZ, CSA-cultivated tea plant YK10, and CSS-cultivated tea plants including SCZ, LJ43, TGY, and HD.

To obtain glycosyltransferase gene family members for our analysis, we employed a multistep approach to ensure the reliability and accuracy of the glycosyltransferase gene family members selected. Initially, we used PF00201 (http://pfam.xfam.org/) as the hidden Markov model (HMM) and HMMSERCH (https://github.com/qinbill/HmSearch) with a stringent *e*-value threshold of 1e−5 to identify UGTs in 28 tea plants. To ensure the reliability of our findings, we performed Batch CD-Search (https://www.ncbi.nlm.nih.gov/Structure/bwrpsb/bwrpsb.cgi) to validate the structural domains retrieved in the search results, excluding genes that lacked discernible PSPG motifs, had sequence lengths below 300 amino acids, or were redundant. Through this comprehensive approach, we obtained a reliable and accurate set of glycosyltransferase gene family members for our analysis. This rigorous procedure enhances the robustness of our findings. Identification of UGT gene family members in 28 plant species is described in Supplementary Method S1.

### Orthogroup analysis of the UGT gene family

For comparison, we used OrthoFinder v2.2.7 (Emms and Kelly 2019) to cluster homologous genes of UGTs from tea plants by sequence similarity, the BLAST bit scores were normalized based on gene length and phylogenetic distance, and the modified BLASTP settings used were as follows: “-seq yes, -soft masking true, -use sw tback” (following Craig et al. 2021). The results of homology classification were determined and are shown in Supplementary Table S6. Orthogroups and expansion/contraction analysis of the UGT gene family in 28 plant species are described in Supplementary Method S2.

### Collinearity analysis of UGTs in tea

“One Step MCScanX” from Tbtools (Chen et al. 2020a) was used to perform comparisons across species (Arabidopsis, cacao, grape, and tea) as well as among tea plant cultivars (DASZ, YK10, and SCZ) to obtain gene pairs and analyze collinear blocks. The ratio of nonsynonymous substitutions (*Ka*) to synonymous substitutions (*Ks*) for these gene pairs was calculated using the “Simple *Ka*/*Ks* Calculator (NG).”

### Phylogenetic analysis of the G group

To determine the phylogenetic relationships among G group (UGT85) genes in plants, protein sequences were aligned with MAFFT, and regions with low-quality alignments were trimmed using trimAl v1.4.rev15 (“-automated1”). A ML species tree was constructed using concatenated gene alignments with IQ-TREE version 1.6.9 with ModelFinder (“-m MFP”) and ultrafast bootstrapping (“-bb 5000”). The protein sequences, aligned proteins, and tree file are reported in Supplementary Data Sets 1 and 2.

ML trees were constructed using the method described above to clarify the phylogenetic relationships among UGT genes in the G group for SCZ (the protein sequences, aligned proteins and tree file is listed in Supplementary Data Sets 3 and 4), as well as these same genes for DASZ, YK10, and SCZ (the protein sequences, aligned proteins, and tree file are listed in Supplementary Data Sets 5 and 6).

### Hypothetical evolutionary histories of G group UGTs in SCZ

After constructing the phylogenetic tree of G group UGTs in SCZ according to the above method, the most parsimonious evolutionary history was inferred. First, a–k branch nodes were established according to the topology of the phylogenetic tree, and the gene/protein sequences under each branch were compared to identify the consistent sequences of each branch. Next, the *Ks* values of the consistent sequences among different branch nodes were calculated, and these branches were inferred to be formed by WGD or TD events of tea plants based on the obtained *Ks* values. When it was not possible to distinguish WGD events or TD events by *Ks* values, we did not label them in the figure. Gene pairs within a branch node were identified as tandem duplicate gene pairs based on the *Ks* values between the gene pairs. Chromosomal localization of UGTs is described in Supplementary Method S3.

### Expression of UGT genes in different tissues and under different types of biotic/abiotic stress

Transcriptome data of UGTs from different tissues as well as under different types of biotic stress (fungal infestation) and abiotic stress (low temperature and drought) in tea plants were obtained from TPIA (Gao et al. 2023). Additionally, gene expression levels during the feeding of *E. obliqua* on tea plants were extracted from published transcriptome data (Jing et al. 2019).

In the expression analysis, the G group was divided into 3 independent clusters, Cluster I, Cluster II, and Cluster III, based on the phylogenetic relationships among G group members in SCZ. The expression levels of UGTs in different tissues of these clusters were visualized using “HeatMap Illustrator” in Tbtools. Heat map colors were calculated using log_2_(TPM + 1) and clustered according to the expression patterns of UGTs. The expression levels of UGTs in Cluster II and Cluster III under different abiotic stresses were visualized using “HeatMap Illustrator” in Tbtools. To compare the responses of UGTs under different levels of stress, the heat map colors were normalized.

### Identification of putatively selected UGTs

To assess whether UGTs have been subjected to artificial selection, θπ, *F*_ST_, and Tajima's *D* were used to detect selective signatures between different populations (wild, cultivator, landrace, and elite populations) (Xia et al. 2020); the data were visualized using Microsoft Excel (2019). The candidate selected UGTs were genes with reduced nucleotide diversity that show significant differences in *F*_ST_ and have Tajima's *D* << 0 compared with the wild population.

### Heterologous expression and purification of recombinant UGT proteins

Total RNA was isolated from tissue samples (1 bud with 2 leaves) from *C. sinensis* var. Shuchazao using the Total RNA Extraction Reagent (Vazyme Biotech Co., Ltd., Nanjing, China) according to the manufacturer's instructions. All primers of *CSS0042294* (UGTH1)*, CSS0018956* (UGT85A53), *CSS0029711* (UGT1), *CSS0020241* (UGT2) and *CSS0030265* (UGT3) for cloning were designed using Primer 5.0 (Supplementary Table S9). The PCR system was established using Prime-STAR MAX DNA Polymerase from Takara, and the full-length coding sequence for candidate genes was amplified from the cDNA (Wang et al. 2020a). The amplified target genes were then ligated into the pGEX-4T1 vector and subsequently sequenced by General Biology (Anhui) Co.

For heterologous expression and purification of recombinant proteins, the recombinant plasmid was introduced into *Escherichia coli* BL21(DE3) cells. The culture was incubated at 37 ℃ until OD_600_ = 0.6 to 0.8; after the culture was cooled to 16 to 18 ℃, isopropyl-β-D-thiogalactopyranoside (IPTG) was added at a final concentration of 1 mm, and protein expression was induced at 16 ℃ for 18 to 20 h. The sediment of the cultures was collected by centrifugation (5000×g, 10 min) and stored at −80 ℃ for at least 2 h. Subsequently, 10 mL of 1× wash buffer was added to resuspend the cultures, and the cells were crushed using an ultrasonic cell crusher (Ningbo Xinzhi, JY92-IIDN) (200 W, ultrasound for 3 s, gap for 1 s, 15 min); the supernatant was obtained by centrifugation (10,000×g, 20 min). The proteins were then purified using GST-binding resin (Novagen, Darmstadt, Germany) following the manufacturer's protocol. The obtained recombinant proteins were loaded onto a HiTrap Q XL anion-exchange column (Cytiva) for anion-exchange purification by fast protein LC (FPLC, BIO-RAD). After washing with 10 mL of buffer A (20 mm Tris, pH 8.0, 20 mm NaCl), the column was eluted with a 20 mL gradient of 20 to 1000 mm NaCl at a flow rate of 0.8 mL/min, and the collected fractions were identified by SDS–polyacrylamide gel electrophoresis (SDS–PAGE) (Supplementary Fig. S15). The target proteins were concentrated and recovered using Amicon Ultra filters (Merck); the purified protein concentration was calculated according to the standard curve (Supplementary Fig. S16). Finally, the purified proteins were stored at −80 ℃ for subsequent analysis.

### Substrate screening of recombinant proteins via an UDP-Glo glycosyltransferase assay

Enzyme assays were performed as previously described (Chen et al. 2020b). Each reaction system (total volume of 25 *μ*L) consisted of Tris–HCl buffer (50 mm, pH = 7.5) and 10 mm dithiothreitol (DTT), along with 2.5 mm UDP-glucose, 2.5 mm of different substrates, and purified protein (3 *μ*g per reaction). The reaction system was incubated in a white, flat-bottomed 384-well enzyme-labeled plate at 30 ℃ for 30 min, and the reaction was terminated by adding a volume of UDP Detection Reagent equal to the reaction system according to the instructions of the UDP-Glo Glycosyltransferase Assay Kit (Promega). After reacting at 25 °C for 30 min, the free UDP produced in the reaction system was detected using a multimode reader (GloMax Explorer, Promega), and the control reaction system contained an equal amount of empty vector protein. At least 3 technical replicates were performed for each reaction. We produced standard curves according to the methods and reagents provided in the UDP-Glo Glycosyltransferase Assay Kit, and the concentration of free UDP generated by the reaction was calculated from the standard curve (Supplementary Fig. S17; *y* = 1222*x* + 3453.8, *R*^2^ = 0.99).

### Identification of the products by LC–MS

The typical assay (200 *μ*L) included 5 mm UDP-glucose, 200 *μ*m substrate, and 10 to 20 *μ*g of purified protein. These enzyme assays were conducted at 30 °C for 3 h. The reaction was stopped and extracted twice with 200 *μ*L of ethyl acetate. Following the evaporation of the organic solvent, the remaining residue was reconstituted in a 50 *μ*L methanol/water (1:1, vol) solution for subsequent LC–MS analysis. LC–MS was carried out using an Agilent ultra-performance LC–MS (UPLC-Q-TOF MS) system. For chromatographic separation, a reversed-phase C18 column (1.8 *μ*m, 100 × 2.1 mm) was used, with the column temperature set at 40 °C and a solvent flow rate of 0.2 mL/min. The sample injection volume was set to 2 *μ*L. Gradient settings and mass spectrometry conditions were set per the laboratory-optimized method (Jing et al. 2020).

### Transient suppression of candidate genes in tea plants using AsODNs

AsODN primers (Supplementary Table S9) for the target genes were designed using an open website (http://www.lifetechnologies.com/se/en/home.html), and the control primer was the positive-sense strand (sODN). The specificity of the primers was verified by BLAST from the TPIA database, and the primer sequences were sent to General Biology (Anhui) Co. for synthesis.

The primers were diluted to 60 *μ*m using deionized water, and the injection method was used for the transient suppression of UGT genes in tea plants according to the following steps. First, 1-year-old tea seedlings (*C. sinensis* var. *sinensis*, ‘Shuchazao’) of the same height and growth status and without pests or diseases were selected. Next, the diluted primers were injected into the dorsal surface of the 1st to 2nd leaves in a dark place (the leaves were injected until they were full according to the size of the tea leaves). The injected tea seedlings were incubated in a greenhouse (25 °C, 60% to 70% humidity) for 24 h, and the samples were collected for subsequent experiments. Specificity validation of AsODN transiently suppressing target genes is described in Supplementary Method S4.

### Fungal infestation after the transient suppression of tea plants

The identified fungal (*Pseudopestalotiopsis camelliae-sinensis*) isolates were incubated on potato dextrose agar (PDA) at 28 °C for 4 to 5 days (Hu et al. 2022); spores from the cultures were suspended in sterile distilled water and crushed using a sterile toothpick (Harteveld et al. 2014), and the spore suspension was adjusted to a concentration of 1 × 10^7^ conidia per ml using a hemocytometer.

Fungal infestation in tea plants was carried out following established protocols (Hu et al. 2022). Leaf surfaces were disinfected with 75% alcohol, rinsed with sterile water, and air-dried before a 50 *μ*L conidial suspension was introduced at 3 puncture sites on the upper leaf surface. Control plants received distilled water. Plastic bags were used to maintain high humidity in the greenhouse (25 to 28 °C, 80% humidity). Four days later, tea plant leaves were photographed using a somatic fluorescence microscope (AXIO Zoom.V16), and the lesion area of tea leaves was determined using ImageJ (https://imagej.net/ij/). Leaves harvested from 2 tea seedlings comprised a single biological replicate. Each experiment included at least 5 biological replicates; materials were collected using liquid nitrogen and stored at −80 °C for subsequent analysis.

### Abiotic stress treatments following the transient suppression of tea plants

Low-temperature treatment was performed using established protocols (Zhao et al. 2020a) in which the transiently suppressed tea plants (AsODN) and the control (sODN) were exposed to 4 ℃ for 48 h, followed by rewarming at 25 ℃ for 1 h. Chlorophyll fluorometry was used to immediately detect the net photosynthetic rate and maximum photochemical efficiency of PSII (*F*_v_/*F*_m_) of tea plants. Smaller *F*_v_/*F*_m_ values indicate greater degrees of damage to tea plants.

For drought treatment, tea plants were treated with 15% (*w/*v) polyethylene glycol 6000 (PEG-6000) solution following a previously described protocol (Jin et al. 2021), and water instead of PEG was applied to the control group. After 24 h, the phenotypic characteristics of the tea leaves were recorded, and the leaf inclination angle of the tea plants was measured using ImageJ software. To determine the water content, the fresh weight (FW) of isolated leaves (1st to 2nd leaves) of the above samples was recorded, and the dry weight (DW) of the leaves was recorded after completely drying the leaves in a drying oven (95 °C). The relative moisture content was calculated using the following formula: (FW-DW)/FW × 100%.

Leaves harvested from 2 tea seedlings comprised a single biological replicate. All the above experiments were performed with at least 3 biological replicates, and the experimental materials were stored at −80 °C after being frozen in liquid nitrogen.

### RNA isolation and qPCR analysis

RNA extraction, reverse transcription quantitative polymerase chain reaction (RT–qPCR), and quantitative real-time PCR (qPCR) were performed according to previously described methods (Wang et al. 2022). The primers used in qPCR are listed in Supplementary Table S9. The glyceraldehyde-3-phosphate dehydrogenase (GAPDH) gene was used as the internal reference gene, and relative gene expression levels were estimated using the 2^−ΔΔCT^ technique (Livak and Schmittgen 2001). Expression levels in the control were standardized to 1, and the experimental data were determined relative to the control.

### Extraction and detection of glycosides and phytohormones in tea samples

Glycosides and phytohormones were extracted and measured using a previously described protocol with slight modifications (Chen et al. 2020b). Specifically, 50 mg of milled samples were extracted with 1 mL of methanol/water/formic acid (15:4:1, *v*/*v*/*v*); the extract was shaken at 4 °C for 60 min, and ultrasonic extraction was performed at 4 °C for 20 min. The supernatant was collected by centrifugation (10,000×g, 20 min, 4 °C) and transferred to a new centrifuge tube. This extraction procedure was repeated 2 more times before the supernatants were combined. The supernatant was concentrated using a vacuum centrifugal concentration system (Eppendorf); next, the supernatant was redissolved using 200 *μ*L of 75% methanol/water solution (containing the internal standard solution of DL-4-chlorophenylalanine at a final concentration of 1.25 ng/mL), passed through a 0.22 *μ*m filter membrane, and placed in the injection vial for LC–MS/MS analysis. Gradient settings and mass spectrometry conditions were set according to the laboratory-optimized method (Jing et al. 2020). The relative concentration of the target compound was calculated using the ratio of the peak area of the target compound to the internal standard (the concentration was 1.25 ng/mL); the relative content of the glycoside or phytohormone was calculated using the following equation: relative content (μg g^−1^) = C × V × 1000^−1^ × m^−1^, where *C* is the relative concentration obtained with the aforementioned reference internal standard, *V* is the resuspension volume (mL), and *m* is the mass of the weighed sample (g). Functional validation of phytohormone to improve resistance in tea plants is described in Supplementary Method S5.

### Statistical analysis

At least 3 independent biological replicates were carried out for all experiments. All measurements were taken in triplicate. Data were expressed as the mean ± Sd from at least 3 biological replicates. All statistical analysis was performed by Student's *t*-tests in GraphPad Prism 8.0. *P* < 0.05 is significant difference, *P* < 0.01 is very significant difference, ns is no significant difference.

### Accession numbers

Sequence data from this article can be found in the Tea Plant Information Archive or GenBank/EMBL data libraries under accession numbers UGTH1 (*CSS0042294*), UGT85A53 (*CSS0018956*), UGT1 (*CSS0029711*), UGT2 (*CSS0020241*, XM_028260042.1) and UGT3 (*CSS0030265*).

## Supplementary Material

koae268_Supplementary_Data

## Data Availability

The data underlying this article are available in the article and in its online supplementary material.

## Supplementary data

The following materials are available in the online version of this article.

**Supplementary Figure S1.** Phylogenetic groupings of plant family UGTs (supports Fig. 1).

**Supplementary Figure S2.** Fungal infestation of tea plants after in vitro spraying with SA, ABA, and JA, respectively (supports Fig. 2).

**Supplementary Figure S3.** LC–MS/MS analyses of enzymatic products formed by UGTH1 (supports Fig. 2).

**Supplementary Figure S4.** Chromosome distribution of UGTs in SCZ (supports Fig. 3).

**Supplementary Figure S5.** Phylogenetic relationships of G group genes in DASZ, YK10, and SCZ (supports Fig. 3).

**Supplementary Figure S6.** Enzymatic activity of recombinant protein with different substrates (supports Figs. 4 and 5).

**Supplementary Figure S7.** Specificity validation of AsODN transiently suppressing UGTs. (Supports Figs. 4 and 5).

**Supplementary Figure S8.** The relative expression of control (sODN) and CsCBFs-suppressed (AsODN) (supports Figs. 4 and 5).

**Supplementary Figure S9.** Specificity validation of AsODN transiently suppressing CBFs (supports Figs. 4 and 5).

**Supplementary Figure S10.** The relative expression of *UGT85A53* after suppressing CsCBFs (supports Fig. 4).

**Supplementary Figure S11.** The relative expression of *UGT1* after suppressing *CsCBF*s (supports Fig. 4).

**Supplementary Figure S12.** Low-temperature and drought stress of tea plants after in vitro spraying with SA, ABA, and JA, respectively (supports Figs. 4 and 5).

**Supplementary Figure S13.** The relative expression of *UGT2* after suppressing *CsCBF*s (supports Fig. 5).

**Supplementary Figure S14.** The relative expression of *UGT3* after suppressing *CsCBF*s (supports Fig. 5).

**Supplementary Figure S15.** Chromatograms and SDS–PAGE gels of recombinant proteins UGTH1, UGT85A53, UGT1, UGT2, and UGT3 purified by anion-exchange chromatography.

**Supplementary Figure S16.** Protein concentration standard curve (supports Figs. 4 and 5).

**Supplementary Figure S17.** The concentration of free UDP standard curve (supports Figs. 4 and 5).

**Supplementary Method S1.** Identification of UGT gene family members in 28 plant species.

**Supplementary Method S2.** Orthogroups and expansion/contraction analysis of the UGTs gene family in 28 plant species.

**Supplementary Method S3.** Chromosomal localization of UGTs in tea.

**Supplementary Method S4.** Specificity validation of AsODN transiently suppressing target genes.

**Supplementary Method S5.** Functional validation of phytohormone to improve resistance in tea plants.

**Supplementary Table S1.** Gene identifiers of candidate UGTs for the plant orthogroups identified by OrthoFinder analysis.

**Supplementary Table S2.** The expansion/contraction ratios for the different UGT phylogenetic groups in each plant species.

**Supplementary Table S3.** The synonymous mutation rate (*Ks*) of UGTs gene pairs in DASZ.

**Supplementary Table S4.** The synonymous mutation rate (*Ks*) of UGTs gene pairs in YK10.

**Supplementary Table S5.** The synonymous mutation rate (*Ks*) of UGTs gene pairs in SCZ.

**Supplementary Table S6.** Gene identifiers of candidate UGTs for different varieties of tea plants.

**Supplementary Table S7.** The expression level of G group gene in different tissue parts of tea plant.

**Supplementary Table S8.** The expression level (TPM) of G group genes in low temperature and drought.

**Supplementary Table S9.** The primers for cloning, transient suppression and qPCR.

**Supplementary Data Set 1.** Sequences and alignment proteins of G group (UGT85) members in plants.

**Supplementary Data Set 2.** Phylogenetic tree file of G group (UGT85) members in plants.

**Supplementary Data Set 3.** Sequences and alignment proteins of G group members in SCZ.

**Supplementary Data Set 4.** Phylogenetic tree file of G group members in SCZ.

**Supplementary Data Set 5.** Sequences and alignment proteins of G group members in SCZ, YK10 and DASZ.

**Supplementary Data Set 6.** Phylogenetic tree file of G group members in SCZ, YK10, and DASZ.

**Supplementary Data Set 7.** Phylogenetic tree file of 28 plant species.

## Dive Curated Terms

The following phenotypic, genotypic, and functional terms are of significance to the work described in this paper:

NPR1 Gramene: TEA005670
NPR1 Araport: TEA005670

PR1 Gramene: TEA004542
PR1 Araport: TEA004542

ABA Gramene: Abscisic acid
ABA Araport: Abscisic acid

UDP Gramene: Uridine diphosphate
UDP Araport: Uridine diphosphate

NHP Gramene: N-hydroxy-pipecolic acid
NHP Araport: N-hydroxy-pipecolic acid
