# Supplementary material for: Evolution and functional divergence of glycosyltransferase genes shaped the quality and cold tolerance of tea plants
Source: Plant Cell. 2024 Oct 4;37(1):koae268. doi: 10.1093/plcell/koae268 (PMC11663605; doi:10.1093/plcell/koae268)
Supplement: koae268_Supplementary_Data [file koae268_supplementary_data.zip › Supplementary Data.pdf]

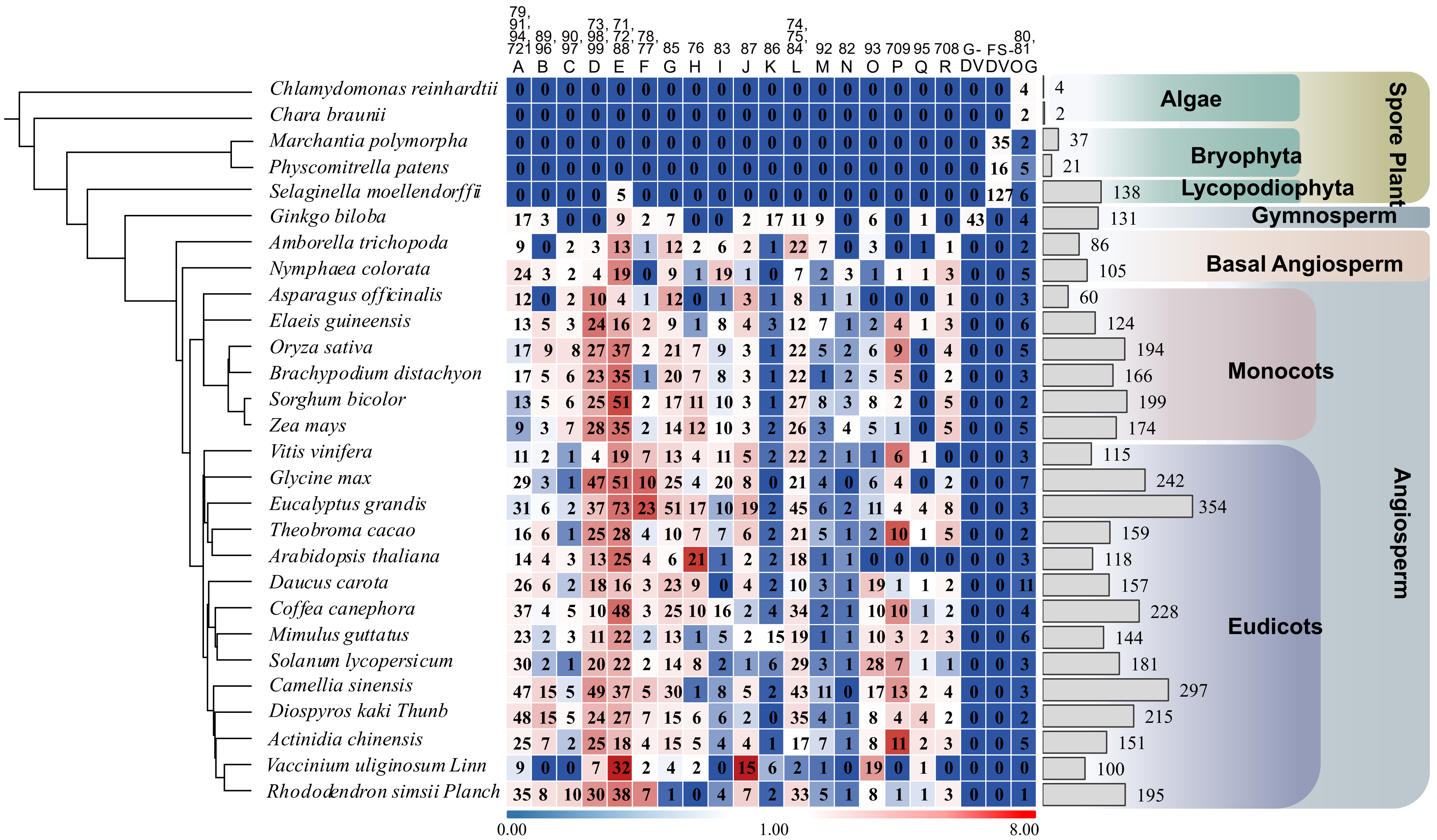

Supplementary Figure S1. Phylogenetic groupings of plant family UGTs. (Supports Figure 1)

The number of UGTs identified for each phylogenetic group is indicated. For each phylogenetic group (i.e. column), the color scale is drawn according to the following ratio: the percentage of UGTs in the phylogenetic group compared with the total UGTs within a given taxon/the percentage of UGTs in the phylogenetic group compared with the total UGTs within the taxon where the group is first identified in. Numbers on the branches represent gains and losses of gene families. G-DV, gymnosperm divergent group; FS-DV, free-sporing (non-seed) plant divergent group; OG, outgroup that can change sterols and lipids, and these enzymes differ from other plant UGTs in that the PSPG motif is less conserved.

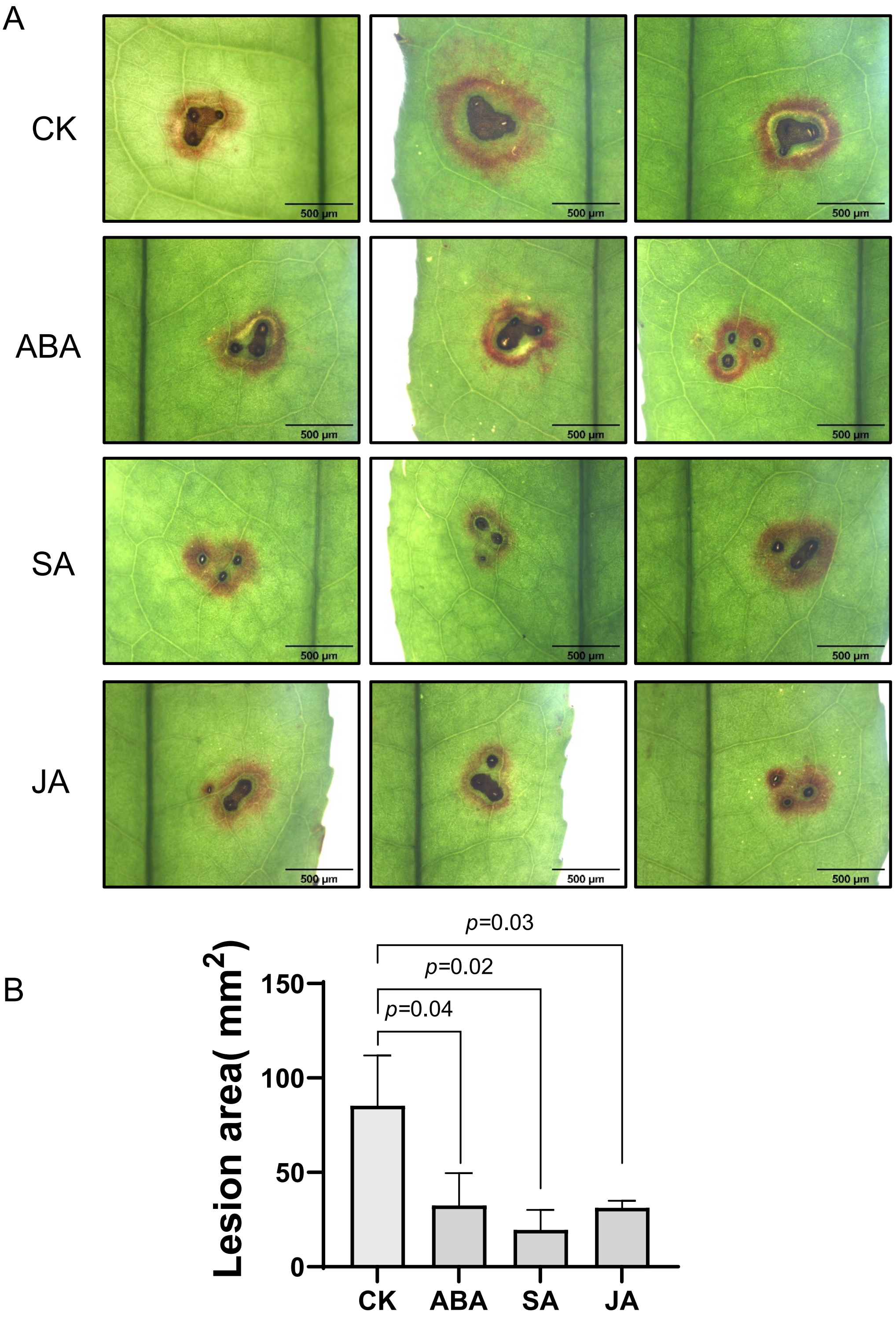

Supplementary Figure S2. Fungal infestation of tea plants after in *vitro* spraying with Salicylic acid (SA), Abscisic acid (ABA) and Jasmonic acid (JA), respectively. (Supports Figure 2)

(A) Disease symptoms were observed under a stereomicroscope after fungal infection for 4 days following hormone spraying. **(B)** Lesion area after fungal infection for 4 days in control (CK) and tea plant after hormone spraying. CK, control. Data were expressed as the mean ± SD from at least three biological replicates. All statistical analysis was performed Student's *t*-tests

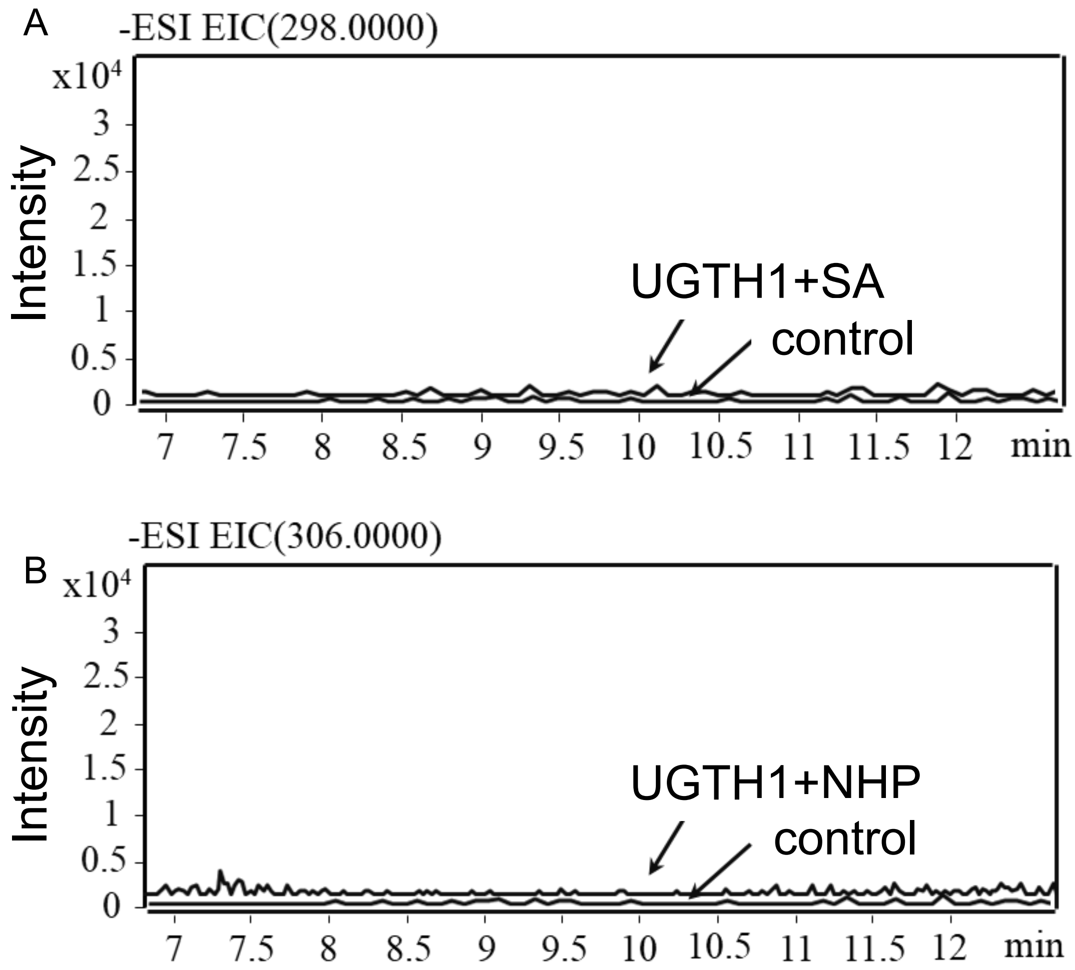

Supplementary Figure S3. LC–MS/MS analyses of enzymatic products formed by UGTH1**.** (Supports Figure 2)
(A) Recombinant UGTH1 has no glycosyltransferase activity to salicylic acid (SA).
(B) Recombinant UGTH1 has no glycosyltransferase activity to N-Hydroxypipecolic acid (NHP).
'control' represents a reaction system containing an equal amount of empty vector protein.

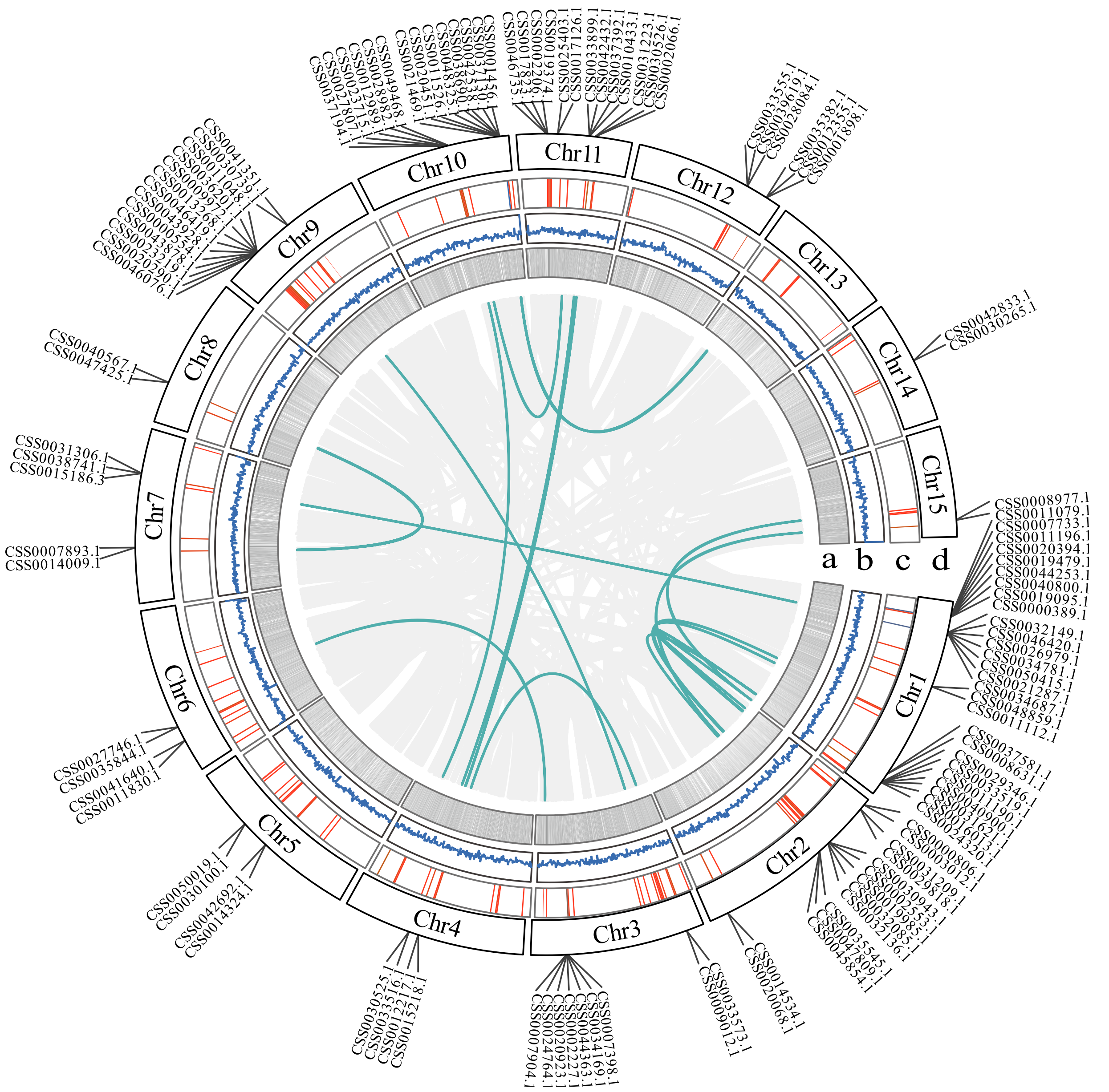

Supplemental Figure S4. Chromosome distribution of UGTs in SCZ. (Supports Figure 3)
Tandem duplication (TD) genes in the UGT family are shown on chromosomes. (a), gene density; (b), GC content; (c), the position of UGTs on chromosomes; (d), chromosome of tea plants.

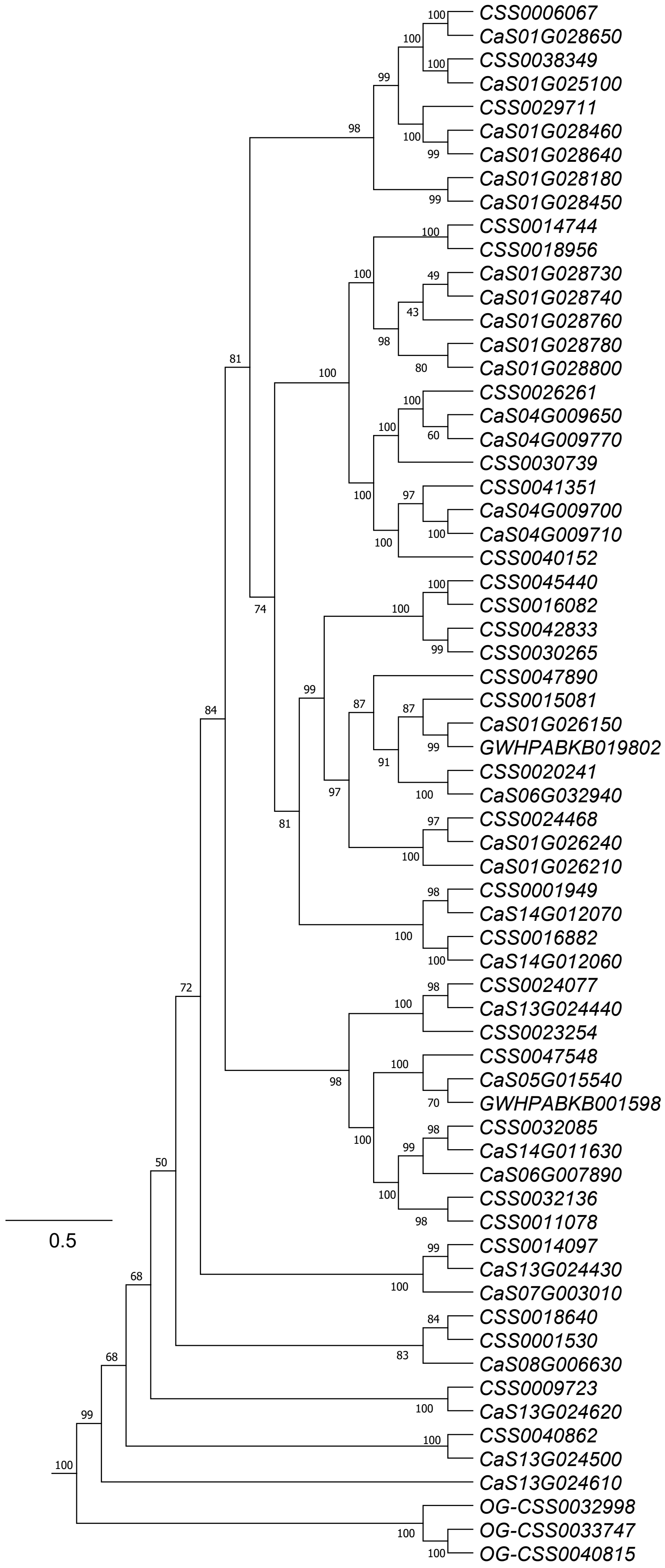

Supplementary Figure S5. Phylogenetic relationships of G group genes in DASZ,YK10 and SCZ**.** (Supports Figure 3)

Phylogenetic relationships were reconstructed using IQ-Tree and the JTT + I + G4 substitution model. Scale bar, the average number of AA substitutions per site.

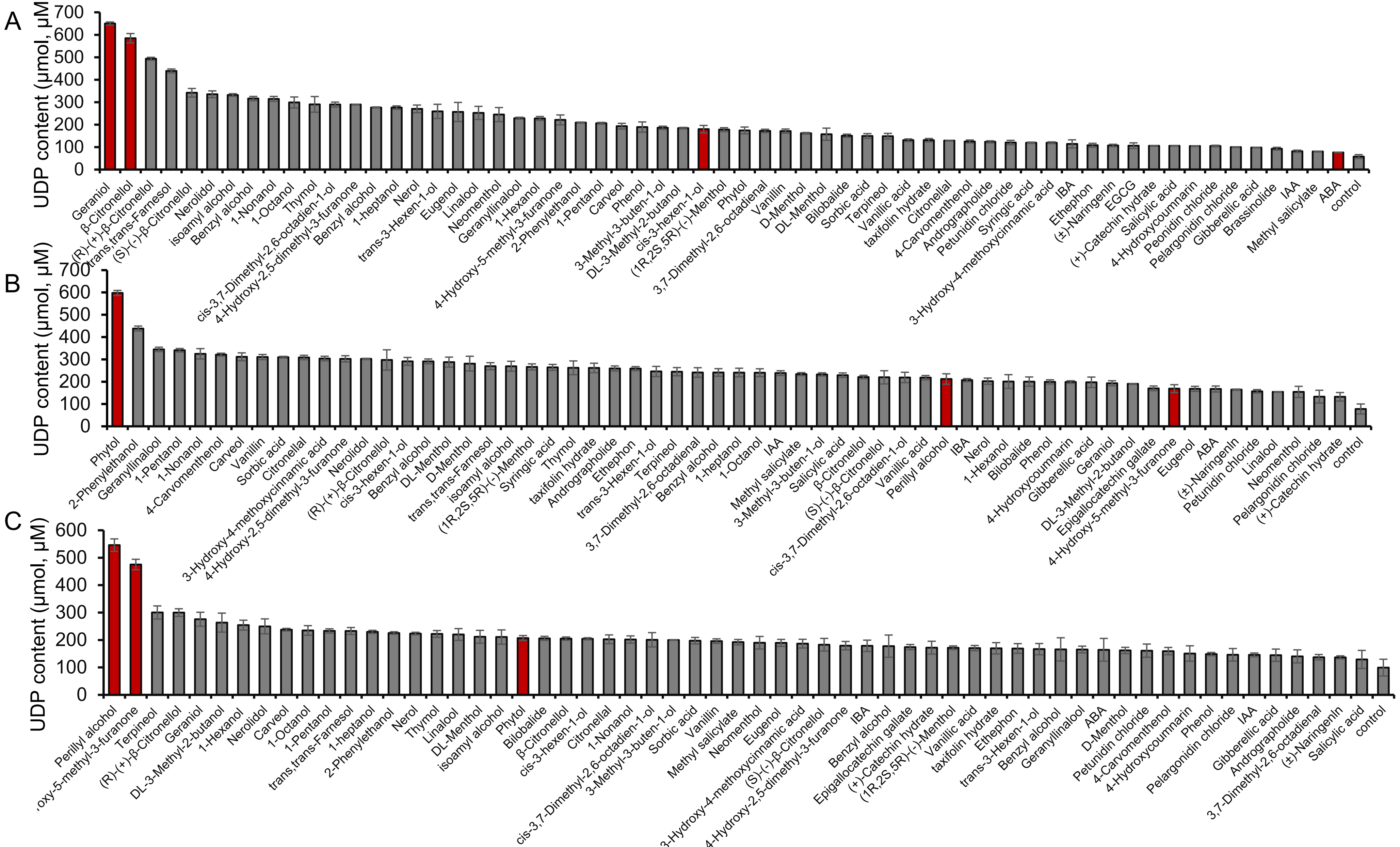

Supplementary Figure S6. Enzymatic activity of recombinant protein with different substrates**.** (Supports Figure 4, Figure 5)
Relative UDP content of recombinant protein towards different substrates was determined by UDP-Glo^{TM} glycosyltransferase assay kit. **(A)** Enzymatic activity of recombinant UGT1 with different substrates. Substrates compared to UGT85A53 are labeled in red. **(B)** Enzymatic activity of recombinant UGT3 with different substrates. Substrates compared to UGT2 are labeled in red. **(C)** Enzymatic activity of recombinant UGT2 with different substrates. Substrates compared to UGT3 are labeled in red. Relative amounts to UDP calculated from standard curves, and data were expressed as the mean±SD from at least three technical replicates.

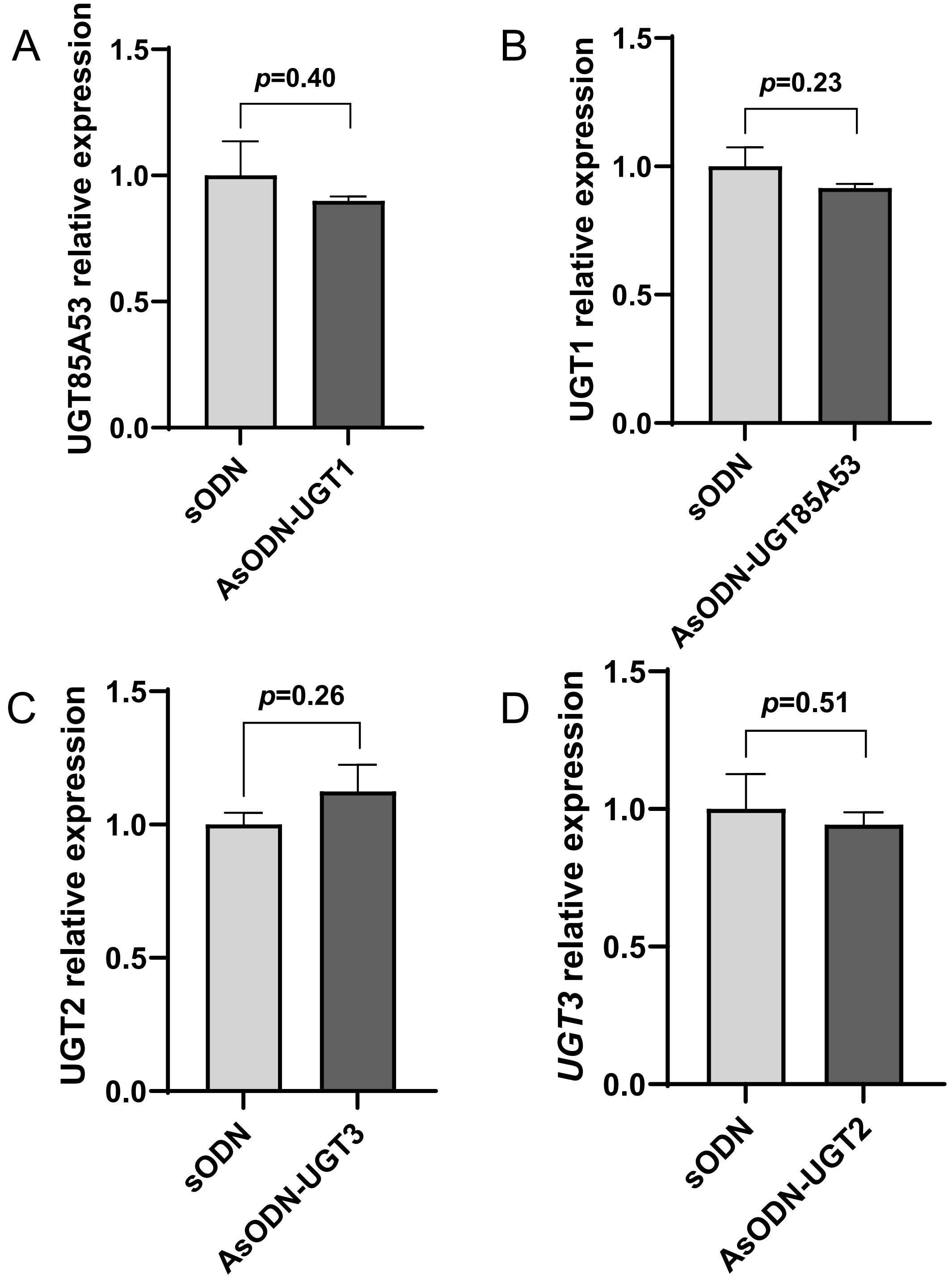

Supplementary Figure S7. Specificity validation of AsODN transiently suppressing *UGT*s**.** (Supports Figure 4, Figure 5)

(A) Relative expression of *UGT85A53* after transient suppression of *UGT1*. **(B)** Relative expression of *UGT1* after transient suppression of *UGT85A53*. **(C)** Relative expression of *UGT2* after transient suppression of *UGT3*. **(D)** Relative expression of *UGT3* after transient suppression of *UGT2*. Data were expressed as the mean ± SD from at least three biological replicates. All statistical analysis was performed by Student's *t*-tests.

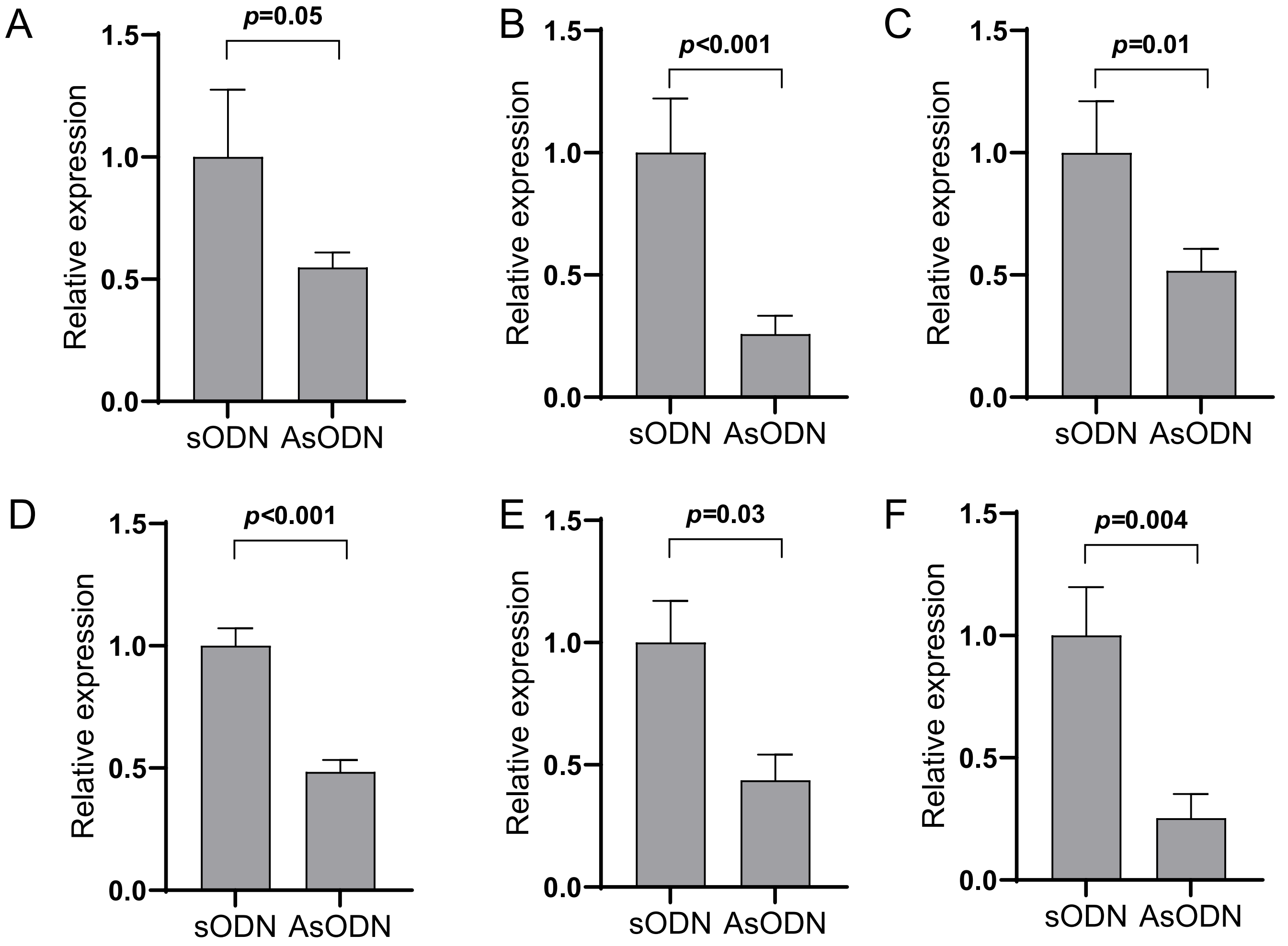

Supplementary Figure S8. The relative expression of control (sODN) and *CsCBFs*-suppressed (AsODN). (Supports Figure 4, Figure 5)

(A)-(F). The relative expression of control (sODN) and *CsCBF1, CsCBF2, CsCBF3, CsCBF4, CsCBF5, CsCBF6* suppressed(AsODN), respectively. Data were expressed as the mean ± SD from at least three biological replicates. All statistical analysis was performed by Student's *t*-tests. The levels in the control were standardized to 1 and the experimental data were relative to this.

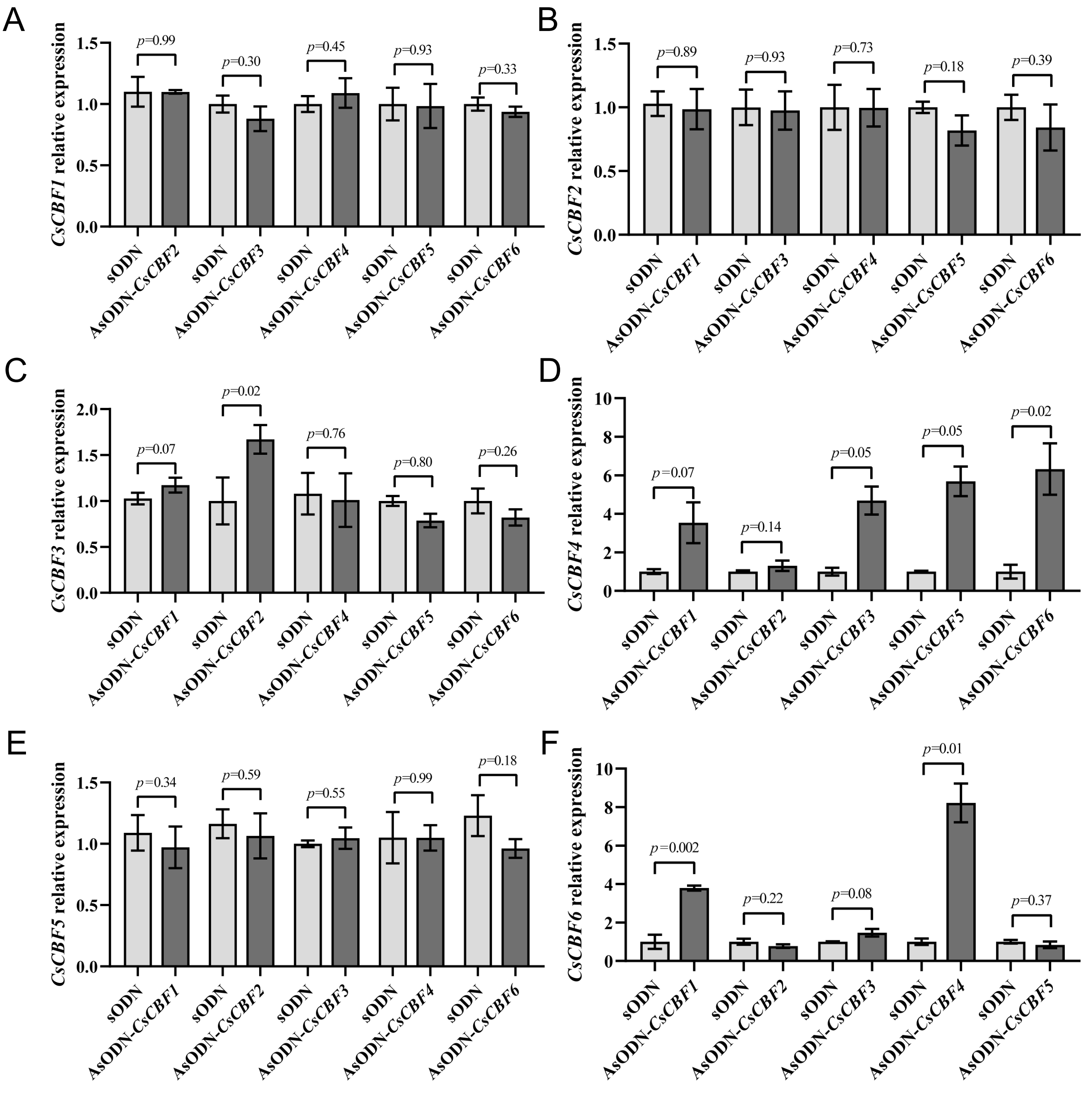

Supplementary Figure S9. Specificity validation of AsODN transiently suppressing *CBF*s**.** (Supports Figure 4, Figure 5)

Relative expression of *CBF1-CBF6* **(A)-(F)** after transient suppression of other *CBF*s. The levels in the control were standardized to 1 and the experimental data were relative to this. Data were expressed as the mean ± SD from at least three biological replicates. All statistical analysis was performed by Student's *t*-tests.

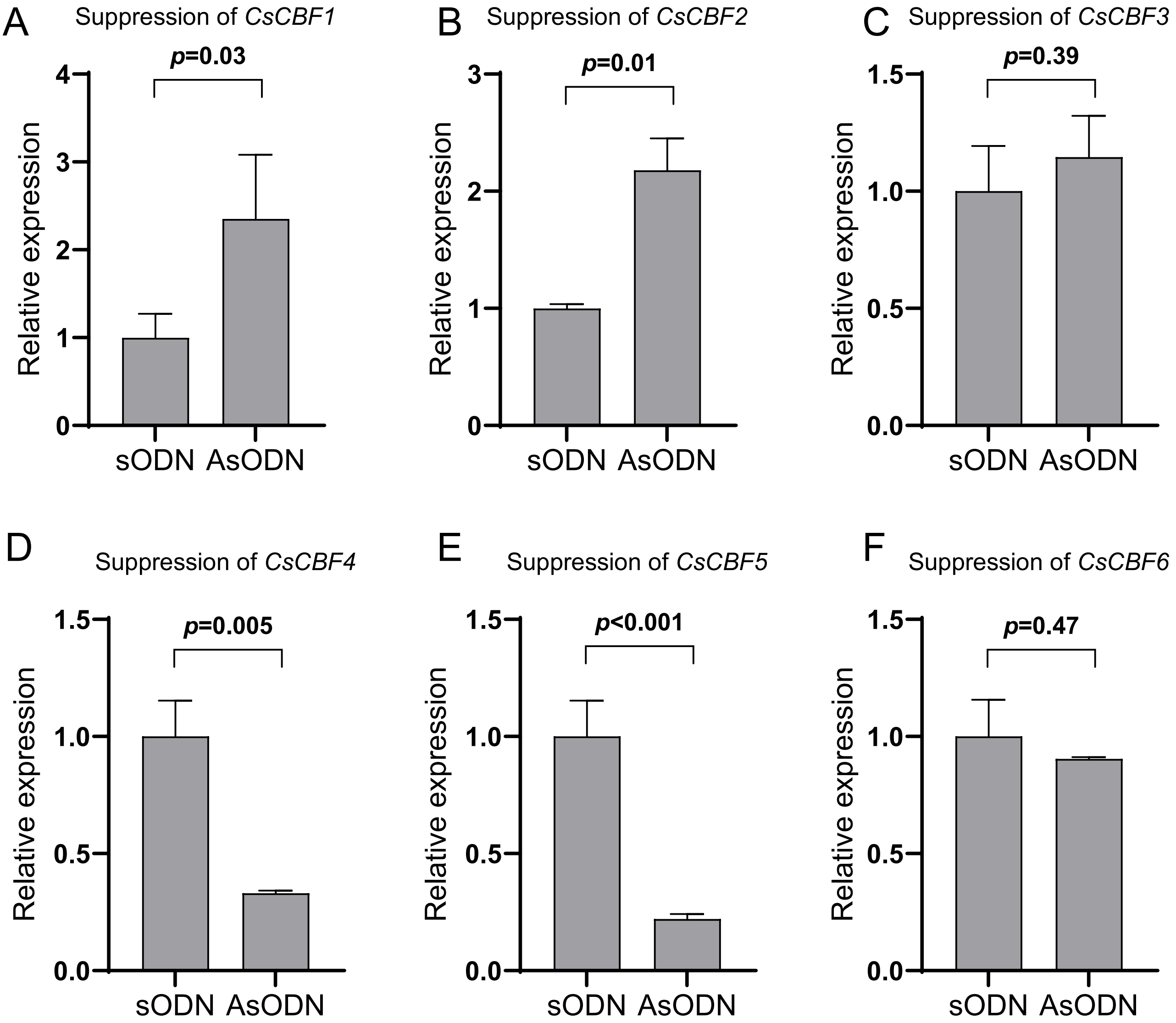

Supplementary Figure S10. The relative expression of *UGT85A53* after suppressing *CsCBFs*. (Supports Figure 4)

(A)-(F). The relative expression of *UGT85A53* after suppressing *CsCBF1, CsCBF2, CsCBF3, CsCBF4, CsCBF5, CsCBF6*, respectively. Data were expressed as the mean ± SD from at least three biological replicates. All statistical analysis was performed by Student's *t*-tests. The levels in the control were standardized to 1 and the experimental data were relative to this.

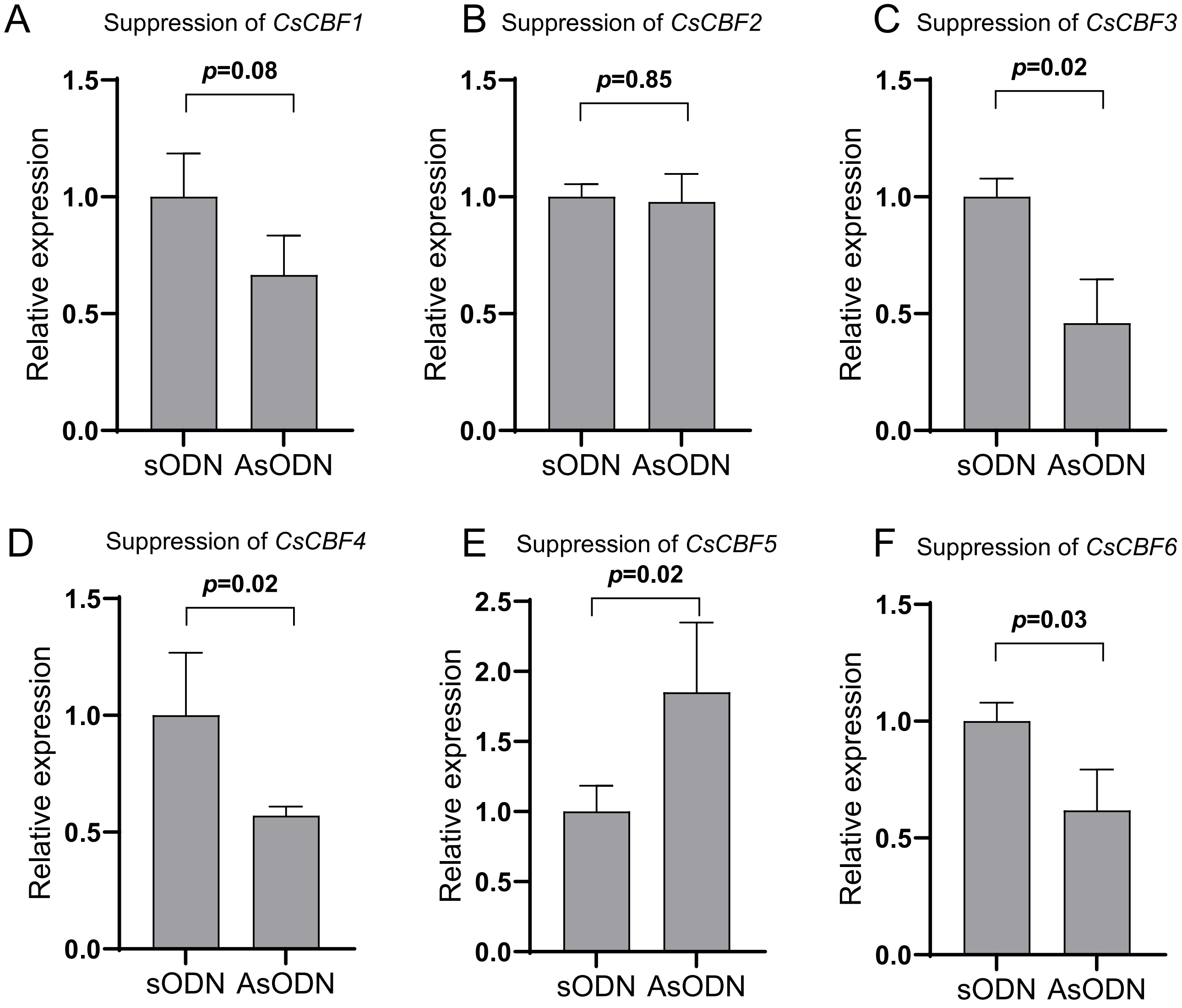

Supplementary Figure S11. The relative expression of *UGT1* after suppressing *CsCBFs*. (Supports Figure 4)

(A)-(F). The relative expression of *UGT1* after suppressing *CsCBF1, CsCBF2, CsCBF3, CsCBF4, CsCBF5, CsCBF6*, respectively. Data were expressed as the mean ± SD from at least three biological replicates. All statistical analysis was performed by Student's *t*-tests. The levels in the control were standardized to 1 and the experimental data were relative to this.

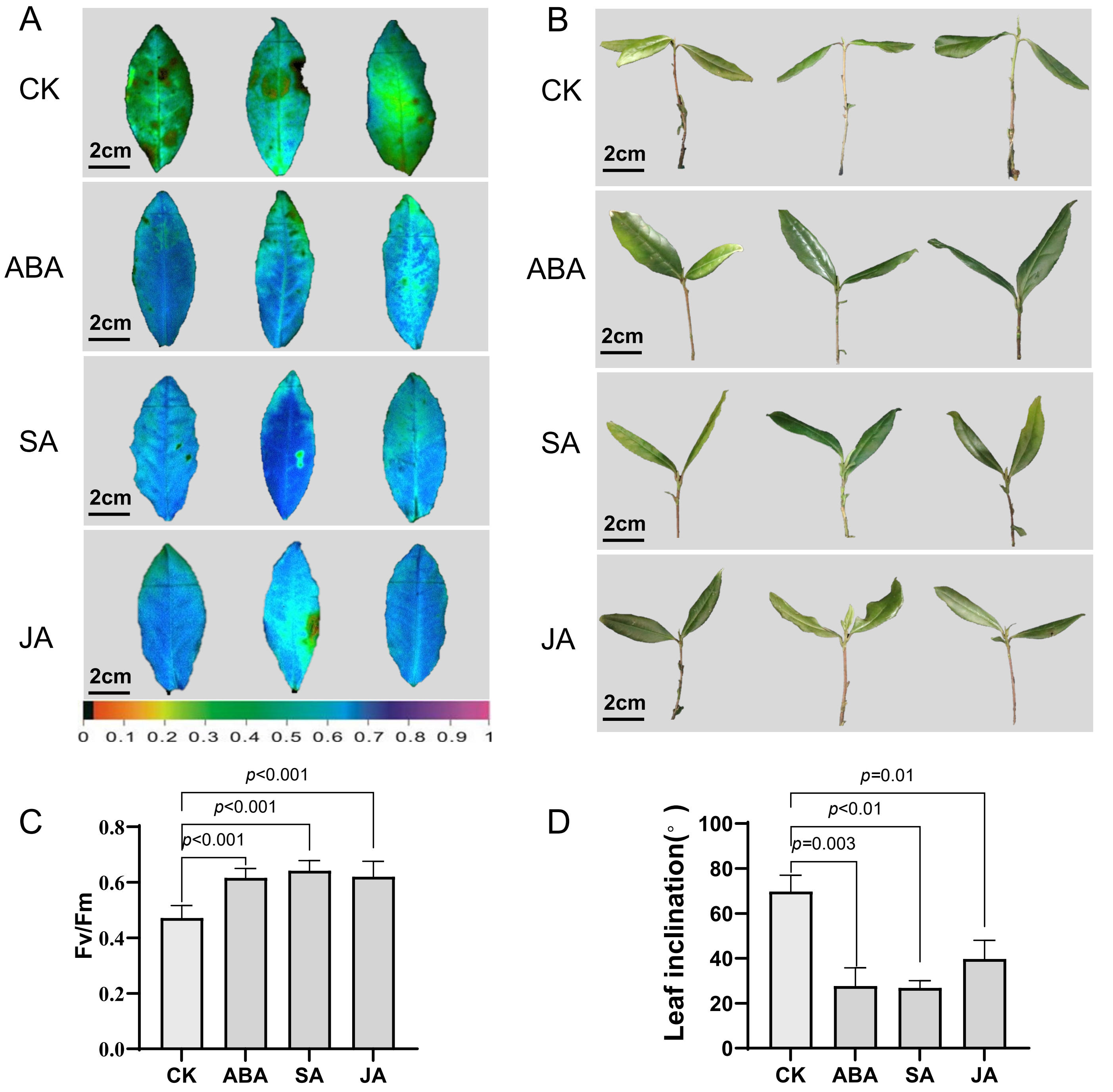

Supplementary Figure S12. Low temperature and drought stress of tea plants after in *vitro* spraying with Salicylic acid (SA), Abscisic acid (ABA) and Jasmonic acid (JA), respectively. (Supports Figure 4, Figure 5)
(A) Chlorophyll fluorescence images of tea plants under 4℃ for 48h following hormone spraying. Purple-blue color indicates normal state of the photosynthetic apparatus, while green and yellow colors indicate damage to photosystem Ⅱ. **(B)** The tea plant phenotypes of drought stress for 24h after hormone spraying, respectively. Images were digitally extracted for comparison. **(C)** Statistical analysis of maximum photochemical efficiency of photosystem II (*Fv/Fm*) in A). **(D)** Leaf inclination of tea plant after drought stress for 24h following hormone spraying, respectively. CK, control. Data were expressed as the mean ± SD from at least three biological replicates. All statistical analysis was performed by Student's *t*-tests. Images in A), B) were digitally extracted for comparison.

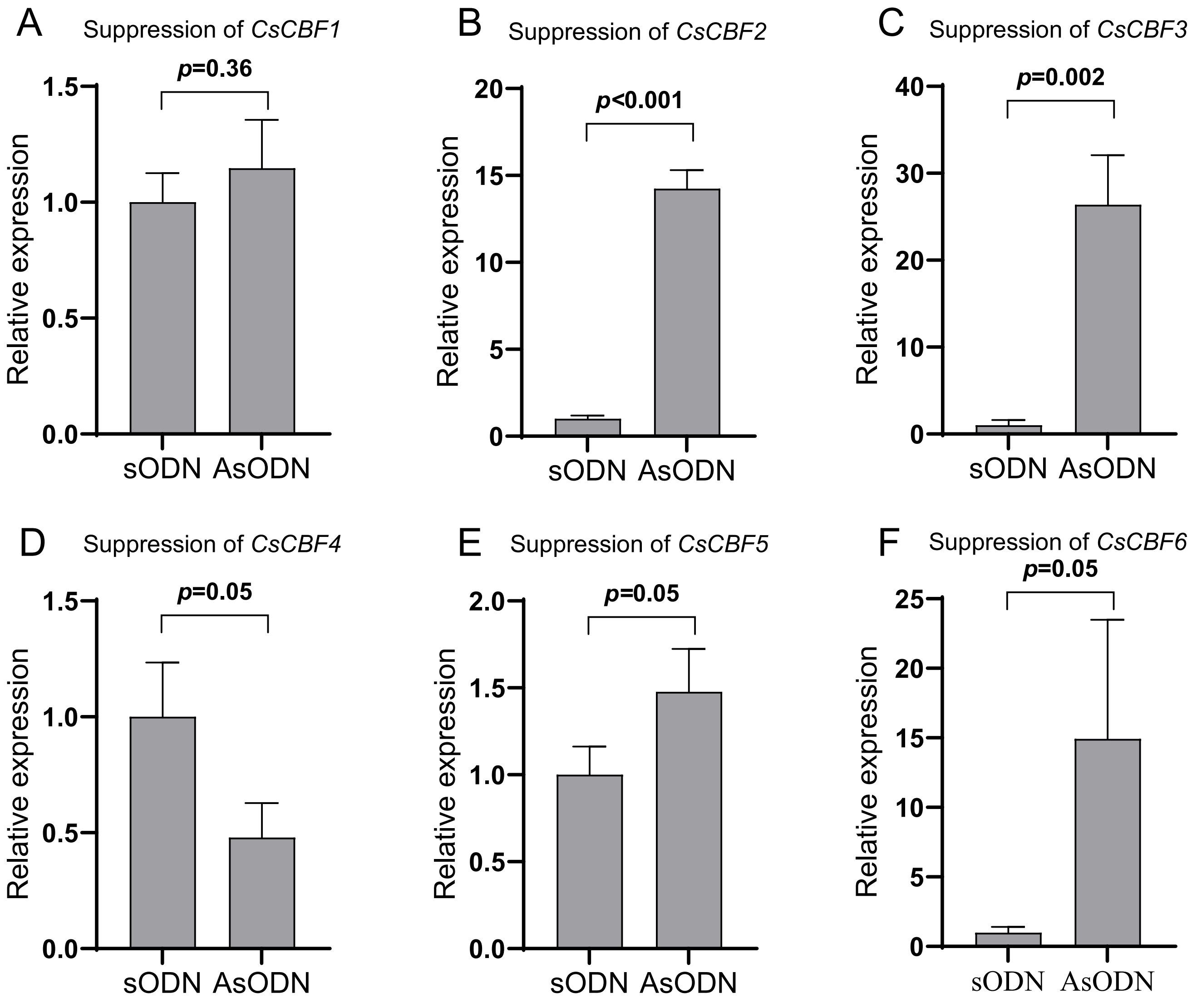

Supplementary Figure S13. The relative expression of *UGT2* after suppressing *CsCBFs*. (Supports Figure 5)

(A)-(F). The relative expression of *UGT2* after suppressing *CsCBF1, CsCBF2, CsCBF3, CsCBF4, CsCBF5, CsCBF6*, respectively. Data were expressed as the mean ± SD from at least three biological replicates. All statistical analysis was performed by Student's *t*-tests. The levels in the control were standardized to 1 and the experimental data were relative to this.

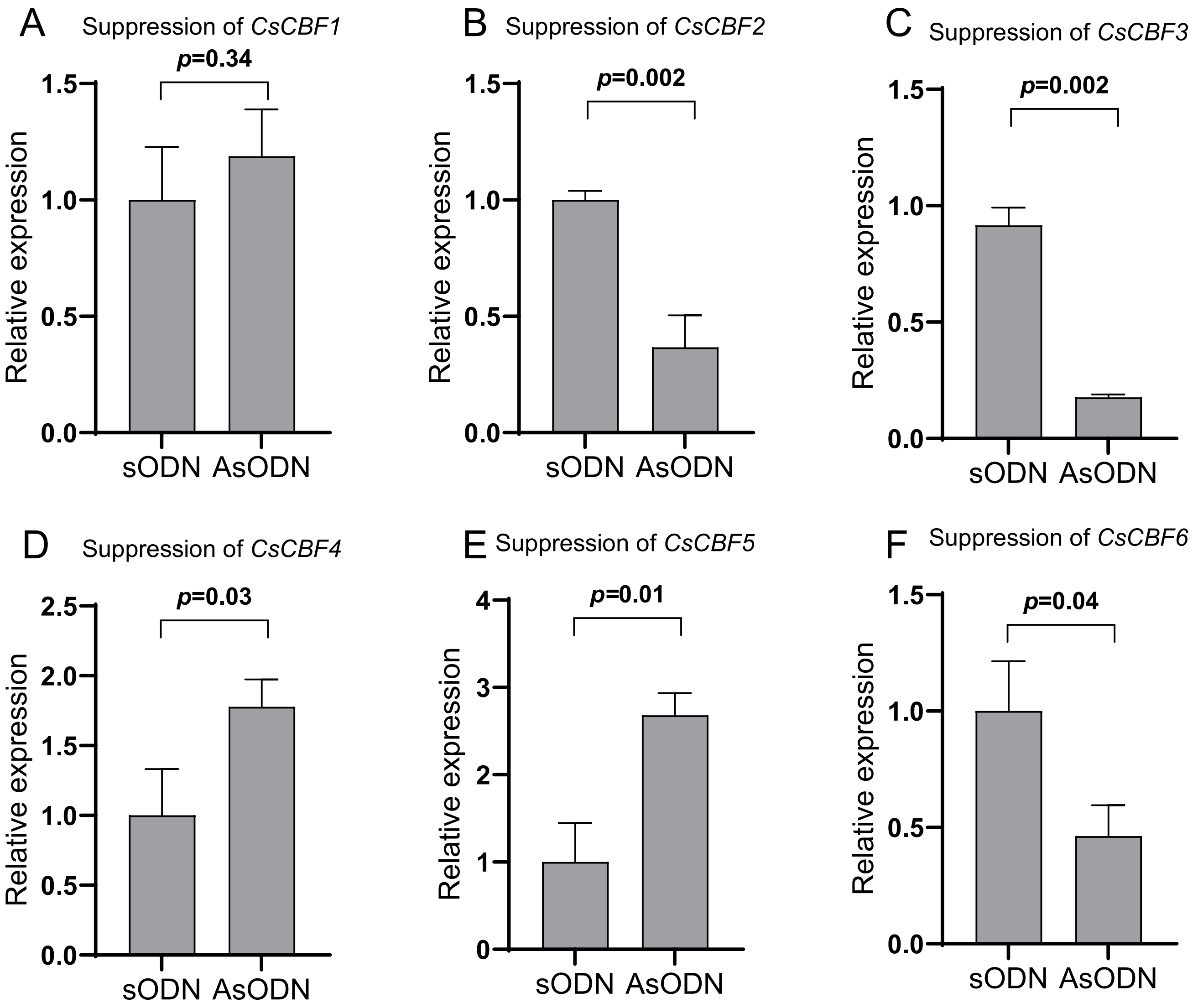

Supplementary Figure S14. The relative expression of *UGT3* after suppressing *CsCBFs*. (Supports Figure 5)
(A)-(F). The relative expression of *UGT3* after suppressing *CsCBF1, CsCBF2, CsCBF3, CsCBF4, CsCBF5, CsCBF6*, respectively. Data were expressed as the mean ± SD from at least three biological replicates. All statistical analysis was performed by Student's *t*-tests. The levels in the control were standardized to 1 and the experimental data were relative to this.

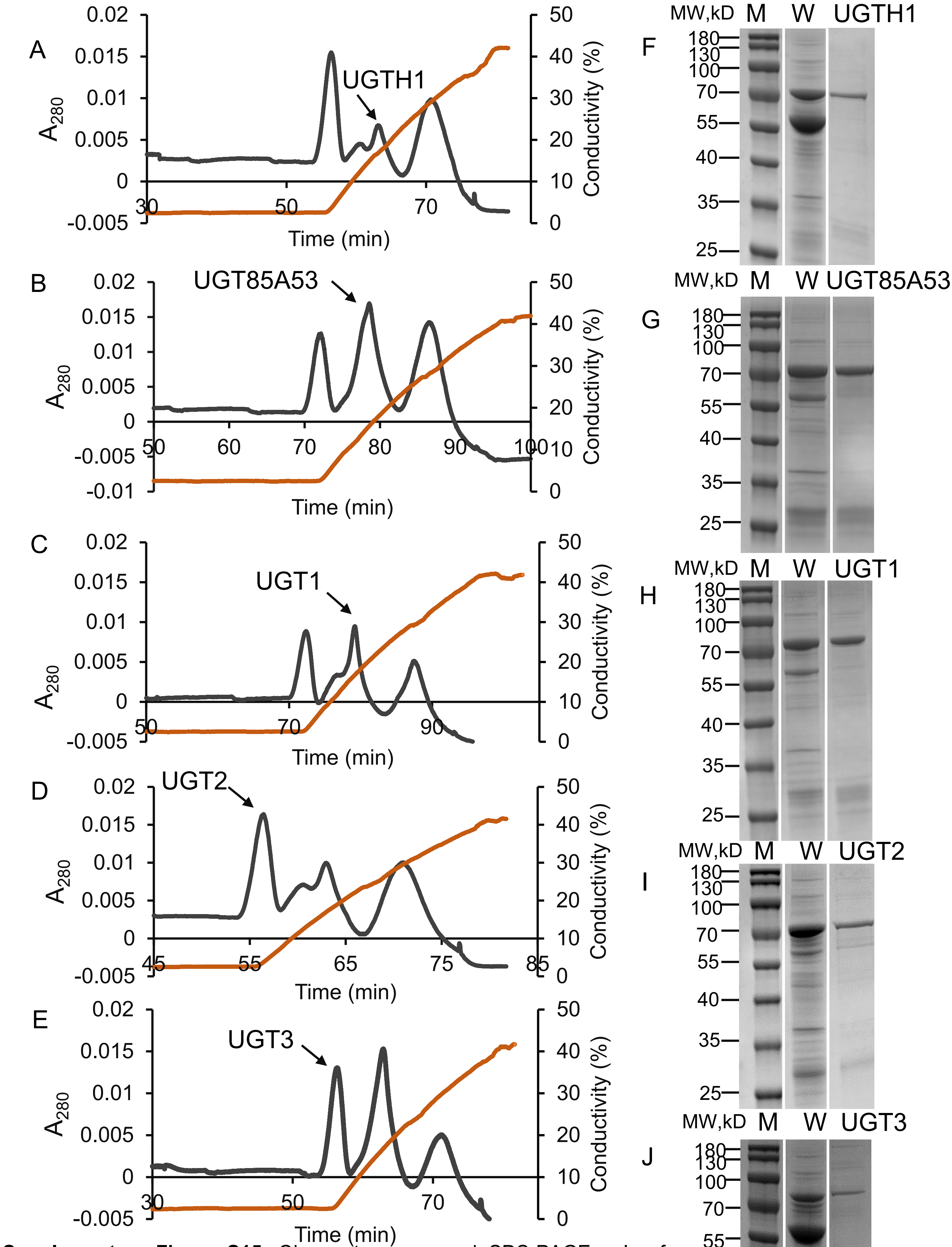

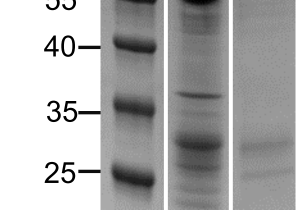

Supplementary Figure S15. Chromatograms and SDS-PAGE gels of recombinant proteins UGTH1 (A, F), UGT85A53 (B, G), UGT1 (C, H), UGT2 (D, I), and UGT3 (E, J) purified by anion-exchange chromatography. (Supports Figure 2, Figure 4, Figure 5)

The red line represents the conductivity curve of anion-exchange chromatography. M, marker; W, mixture of recombinant proteins.

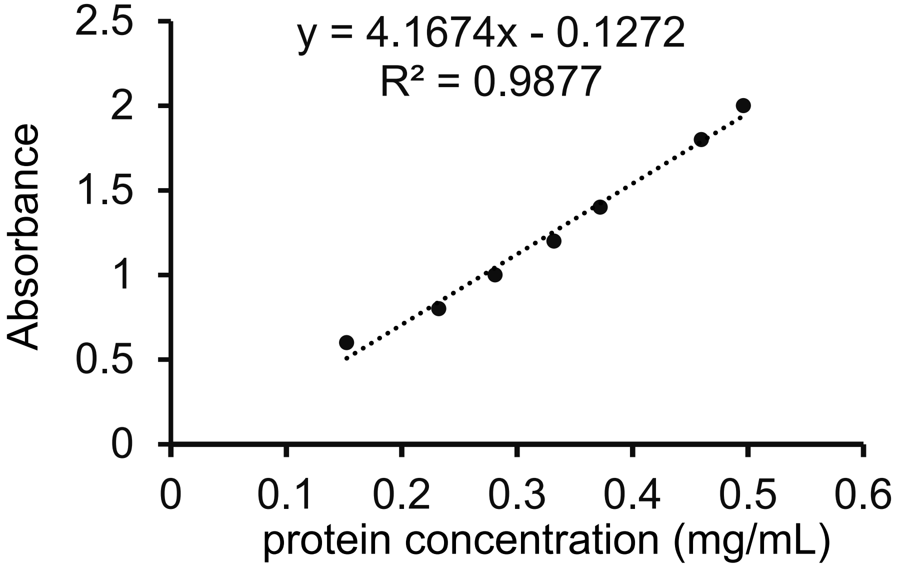

Supplementary Figure S16. Protein concentration standard curve.
(Supports Figure 4, Figure 5)
The dotted line represents the fitted curve.

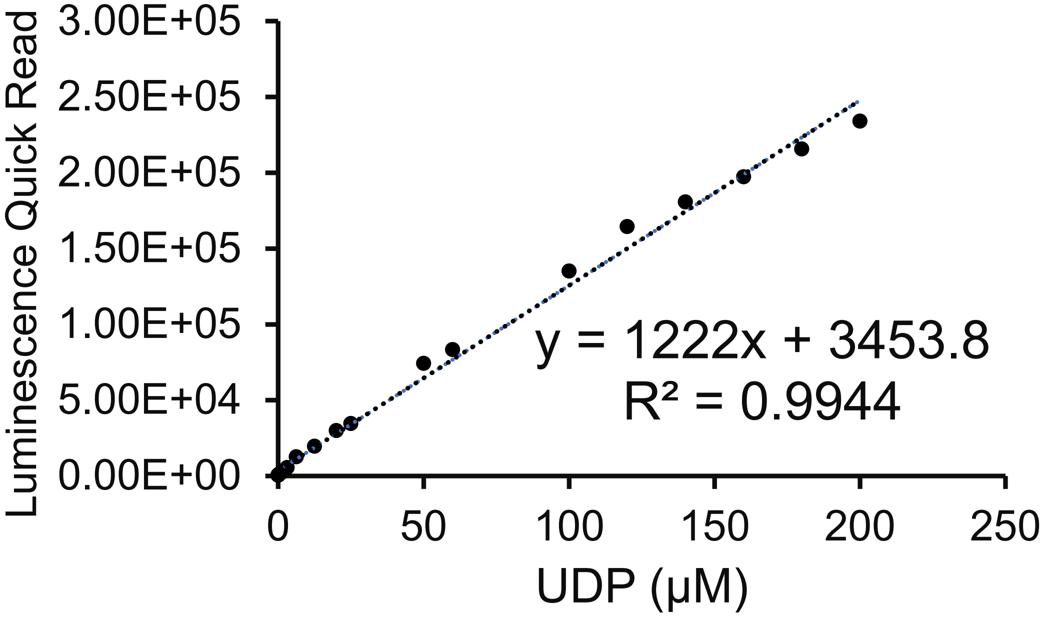

Supplementary Figure S17. The concentration of free UDP standard curve. (Supports Figure 4, Figure 5)
The dotted line represents the fitted curve.

Supplementary Methods

Supplementary Method S1. Identification of UGT gene family members in 28 plant species

Genome sequences of 28 plant species from public plant databases were downloaded to facilitate our study, including NCBI (https://www.ncbi.nlm.nih.gov/): *Actinidia chinensis*, *Rhododendron simsii*, *Elaeis guineensis*, *Asparagus officinalis*; PhytozomeV13 (https://phytozome.jgi.doe.gov/pz/portal.html): *Chlamydomonas reinhardtii*, *Physcomitrella patens*, *Selaginella moellendorffii*, *Marchantia polymorpha*, *Amborella trichopoda*, *Nymphaea Linn*, *Vitis vinifera*, *Glycine max*, *Theobroma cacao*, *Arabidopsis thaliana*, *Mimulus guttatus*, *Solanum lycopersicum*; Ensembl Plants (https://plants.ensembl.org/info/website/ftp/index.html): *Oryza sativa*, *Zea mays*, *Sorghum bicolor*, *Brachypodium distachyon*, *Eucalyptus grandis*, *Daucus carota*, *Diospyros kaki Thumb*; OrcAE (https://bioinformatics.psb.ugent.be/orcae/): *Chara braunii*; PlantGDB (http://www.plantgdb.org/): *Ginkgo biloba*; GDV (https://www.vaccinium.org/): *Vaccinium uliginosum Linn*. Coffee Genome Hub (http://coffee-genome.org/): *Coffea canephora*; TPIA (http://tpia.teaplants.cn/): *Camellia sinensis* (DASZ, YK10, SCZ, LJ43, TGY, HD).

To obtain the glycosyltransferase gene family members for our analysis, we employed a multi-step approach, aiming to ensure the reliability and accuracy of the glycosyltransferase gene family members selected. Initially, we employed PF00201 (http://pfam.xfam.org/) as the Hidden Markov Model (HMM) and utilized HMMSERCH (https://github.com/qinbill/HmSearch) with a stringent e-value threshold of 1e-5 to identify UGTs in 28 species. To ensure the reliability of our findings, we performed Batch CD-Search (https://www.ncbi.nlm.nih.gov/Structure/bwrpsb/bwrpsb.cgi) to validate the structural domain on the search results, excluding genes that lacked discernible PSPG motifs, exhibited sequence lengths below 300 amino acids, or were redundant in nature. By implementing this comprehensive approach, we strived to obtain a reliable and accurate set of glycosyltransferase gene family members for our analysis. This rigorous process enhances the credibility of our findings and ensures the integrity of our research.

Supplementary Method S2. Orthogroups and expansion/contraction analysis of the UGTs gene family in 28 plant species

For comparison, we used OrthoFinder v2.2.7 (Emms and Kelly, 2019) to cluster homologous genes of UGTs from all 28 species by sequence similarity, the BLAST bit scores were normalized considering gene length and phylogenetic distance, and the modified BLASTP options "-seq yes, -soft masking true, -use sw tback" (following (Craig et al., 2021)). For phylogenetic analysis, protein sequences from orthogroups containing a single gene in all 28 included species (i.e., putative single copy orthologs) were aligned with MAFFT and trimmed for regions of low-quality alignment using trimAl v1.4.rev15 ("-automated1") (Capella-Gutierrez et al., 2009). A maximum likelihood (ML) species tree (Supplementary Dataset S7) was constructed using concatenated gene alignments with IQ-TREE version 1.6.9 (Nguyen et al., 2015), running with ModelFinder ("-m MFP") and ultrafast bootstrapping ("-bb 5000"). And calibrate the ML species trees obtained with reference to the literature (Leebens-Mack et al., 2019). The number of genes falling within each homology classification was determined, while genes that could not be identified as belonging to specific subfamilies underwent homology matching on NCBI for classification anchoring. The results of homology classification were determined and presented in Supplementary Table S1.

To compute the expansion/contraction ratios and visualize the distribution of UGTs, we created a color scale based on relevant literature (Wilson and Tian, 2019). The percentage of UGTs in the phylogenetic group is compared with the total UGTs within a given taxon/the percentage of UGTs in the phylogenetic group compared with the total UGTs within the taxon where the group is first identified in (Supplementary Table S2). A ratio of 1 signifies that there is neither expansion nor contraction of UGTs within a particular taxonomic unit.

Supplementary Method S3. Chromosomal localization of UGTs in tea

The locations of UGTs genes on chromosomes were retrieved from the Tea Plant Information Archive (Xia et al., 2019). To visualize the chromosomal distribution, we employed "Advanced Circos" from Tbtools(Chen et al., 2020) to map the UGTs genes

on the respective chromosomes.

Supplementary Method S4. Specificity validation of AsODN transiently suppressing target genes

To confirm the selectivity of the AsODN method for reducing transcriptional levels of target genes, we performed cross real-time PCR on the cDNAs of all transiently suppressed samples to ensure that the suppression of a *CBF* did not reduce the expression levels of other *CBF*s or that the suppression of a *UGT* did not reduce the expression levels of other *UGT*s. The glyceraldehyde-3-phosphate dehydrogenase (GAPDH) gene was used as the internal reference gene, and relative gene expression was estimated using the $2^{-\Delta\Delta CT}$ technique (Livak and Schmittgen, 2001). The levels in the control were standardized to 1 and the experimental data were relative to this.

Supplementary Method S5. Functional validation of phytohormone to improve resistance in tea plants

To verify the effect of phytohormone on improving tea resistance, we conducted spraying experiments on tea plants according to the following steps: ABA, JA and SA were diluted to 1 mM in deionized water, and Tween 20 was added to the solution at a final concentration of 0.1%. One-year-old healthy tea seedlings (*C. sinensis* var. *sinensis*, 'Shuchazao') were obtained in January 2024 at Dechang Seedling Company (Luan, Anhui, China), and tea leaves and the backs of tea leaves were sprayed until water droplets flowed down, and the control was sprayed with 0.1% Tween 20. The spraying was repeated once after 24 h. The sprayed tea seedlings were incubated in the greenhouse for 48 h for subsequent treatments. Fungal infestation, low temperature and drought treatments are described in the Methods of the manuscript.

Reference

Capella-Gutierrez, S., Silla-Martinez, J.M., and Gabaldon, T. (2009). trimAl: a tool for automated alignment trimming in large-scale phylogenetic analyses. Bioinformatics **25,** 1972-1973.

Chen, C., Chen, H., Zhang, Y., Thomas, H.R., Frank, M.H., He, Y., and Xia, R. (2020). TBtools: An Integrative Toolkit Developed for Interactive Analyses of Big Biological Data. Mol Plant **13,** 1194-1202.

Craig, R.J., Hasan, A.R., Ness, R.W., and Keightley, P.D. (2021). Comparative genomics of Chlamydomonas. The Plant Cell **33,** 1016-1041.

Emms, D.M., and Kelly, S. (2019). OrthoFinder: phylogenetic orthology inference for comparative genomics. Genome Biology **20**.

Leebens-Mack, J., Wickett, N., Deyholos, M.K., Degironimo, L., and Pires, J.C. (2019). One thousand plant transcriptomes and the phylogenomics of green plants. Nature **574**.

Livak, K.J., and Schmittgen, T.D. (2001). Analysis of relative gene expression data using real-time quantitative PCR and the 2(-Delta Delta C(T)) Method. Methods **25,** 402-408.

Nguyen, L.-T., Schmidt, H.A., von Haeseler, A., and Minh, B.Q. (2015). IQ-TREE: A Fast and Effective Stochastic Algorithm for Estimating Maximum-Likelihood Phylogenies. Molecular Biology and Evolution **32,** 268-274.

Wilson, A.E., and Tian, L. (2019). Phylogenomic analysis of UDP-dependent glycosyltransferases provides insights into the evolutionary landscape of glycosylation in plant metabolism. Plant J **100,** 1273-1288.

Xia, E.H., Li, F.D., Tong, W., Li, P.H., Wu, Q., Zhao, H.J., Ge, R.H., Li, R.P., Li, Y.Y., Zhang, Z.Z., Wei, C.L., and Wan, X.C. (2019). Tea Plant Information Archive: a comprehensive genomics and bioinformatics platform for tea plant. Plant Biotechnol J **17,** 1938-1953.
