# Supplementary material for: Evolution and functional divergence of glycosyltransferase genes shaped the quality and cold tolerance of tea plants
Source: Plant Cell. 2024 Oct 4;37(1):koae268. doi: 10.1093/plcell/koae268 (PMC11663605; doi:10.1093/plcell/koae268)
Supplement: koae268_Supplementary_Data [file tpc.11127.2024-s07.pdf › tpc.11127.2024-s07.pdf]

THE PLANT CELL

Evolutionary amplification and functional divergence of UDP-dependent glycosyltransferases genes shapes the quality traits and cold tolerance of tea plants

Jingming Wang, Yutong Hu, Danyang Guo, Ting Gao, Tianqi Liu, Jieyang Jin, Mingyue Zhao, Keke Yu, Wei Tong, Honghua Ge, Yuting Pan, Mengting Zhang, Mengqian Lu, Tingting Jing, Wenkai Du, Xiaoyan Tang, Chenjie Zhao, Wei Zhao, Zhijie Bao, Wilfried Schwab, Enhua Xia, and Chuankui Song

Review Timeline:

Submission Date:	24-May-2024
Editorial Decision:	22-Jun-2024
Resubmission Received:	23-Jul-2024
Editorial Decision:	11-Aug-2024
Revision Received:	22-Aug-2024
Accepted:	27-Aug-2024

Prof. Dr. Chuankui Song
Anhui Agricultural University
State Key Laboratory of Tea Plant Biology and Utilization
Hefei, Anhui
China

Dear Dr. Song:

Thank you for choosing to send your manuscript entitled "Evolutionary amplification and functional divergence of UDP-dependent glycosyltransferases genes shapes the quality traits and cold tolerance of tea plants" for consideration at The Plant Cell. Your submission has been evaluated by members of the editorial board as well as expert reviewers in your field, and we regret to inform you that we are not able to recommend the publication of this manuscript in its current form. We have had input from multiple scientists and we have solicited post-review comments.

During the post-review consultation session, we also agreed that if you could address the major points raised by the reviewers by new experiments, we would welcome a resubmission. Both reviewers find the manuscript greatly improved, but both expressed deep concern about the poor purity of the recombinant UGT proteins resulting in inaccurate and uninterpretable estimations of GT activity. This is a fatal flaw for the current manuscript, but as described by reviewer #2 the problem would be resolved with protein purification and reanalysis of GT activity. We hope you will be able to do these experiments and resubmit to TPC.

If you choose to do so, the resubmitted manuscript may be treated as a new submission, but we would attempt to use at least some of the same reviewers. Nevertheless, reviewers will be asked to assess as a new manuscript (i.e. are the claims fully supported by the data and do the results presented move the field forward?), and not only whether previous reviewer comments have been addressed.

It will be important to convince the editors and reviewers that the study adds significant new understanding(s) of mechanisms or processes, or otherwise represents a significant advance in the field, and that the major claims made are fully justified by the data presented. This includes careful consideration and explanation of the various controls used in experiments, the extent and manner of replication, and the statistical analyses used. Sampling methods and the nature of "biological replicates" should be described precisely (i.e. different plants, parts of plants, pooled tissue, independent pools of tissue, sampled at different times, etc.), along with a clear description of and rationale for any statistical analyses conducted. The reader should know exactly what was sampled; what forms the basis of the calculation of any means and other statistical variables and parameters reported. This is also necessary to ensure that proper statistical analysis was conducted.

The Plant Cell now requires authors to complete and submit an author revisions checklist upon submission of a revised manuscript. The aim of the checklist is to aid authors in preparing a high-quality manuscript, facilitate the review and assessment of revised manuscripts, and help to ensure that journal standards are maintained across the board. If your manuscript is accepted, the completed checklist will be published as supplemental material attached to the article online. Please download a copy of the checklist (a fillable PDF form) at this link, for submission with your revised manuscript: https://tpc.msubmit.net/html/Author_Revisions_Checklist.pdf.

Note also that supplemental materials should be restricted to large datasets and tables, presentation of replicates, and validation of reagents, methods, or genotypes. Any data that are used to support the main claims must be in the main manuscript. Supplemental figure legends must indicate what figure in the main manuscript is supported by the supplemental data presented.

We thank you for your interest in and support of The Plant Cell. We hope we will be able to render a more positive decision on future work.

On behalf of the editorial board,

James Leebens-Mack, Board of Reviewing Editors
Daniel Kliebenstein, Senior Editor
Blake Meyers, Editor-in-Chief

The Plant Cell

-- Reviewer comments:

Reviewer #1 (Comments for the Author):

The authors have made extensive revisions to the manuscript, significantly improving its clarity and the reliability of the data.

Reviewer #2 (Comments for the Author):

This is a substantially revised version of a manuscript in which the authors investigate the rapid evolutionary divergence of the G and H subfamilies of UGTs in two tea species. In my previous review, I asked the authors to provide more information about the proteins that they were using for enzymology and to demonstrate specificity of the antisense oligonucleotides that were being used for target knockdown experiments. While the manuscript has been substantially improved from the previous submission, I still have some considerable reservations about the protein biochemistry that was conducted in this manuscript, which is an important part of the underlying story. I will detail my comments below.
Major comments
• In my previous review, I had asked the authors to demonstrate that AsODNs were specifically targeting the desired genes, and it appears that they have provided these important controls in the revised manuscript.
• In my previous review, I asked to see gels of the purified glycosyltransferases. These gels have been provided in the revision. None of these proteins are actually pure and the authors are assuming that they have produced the correct protein simply by estimating the molecular weight of each recombinant protein on SDS-PAGE gels. These observations are problems for a few reasons: 1) the authors refer to these proteins throughout the manuscript as "purified", and they are not, 2) any effort to standardize these recombinant proteins on a protein mass basis (i.e. 3 micrograms of protein per reaction, etc) is not valid because the proteins are not pure, and 3) because many of the purification gels look similar, it is hard to discern whether some of the measured activities arise from these GTs or E. coli GTs that are consistently co-purifying. To be confident that the ascribed proteins are contributing to the ascribed activities, the authors need to purify these proteins to homogeneity. This could readily be accomplished by performing a secondary anion exchange or size exclusion purification on an FPLC or installing a his-tag on the previous recombinant proteins to perform Glutathione- nickel NTA sequential purifications. Minimally, the authors could only refer to these proteins as partially purified throughout the manuscript and perform mass spectrometry analysis of dissected bands from the SDS-PAGE gel to show that the indicated bands are actually the GT of interest.

Dear Reviewers,

I hope this letter finds you in good health and high spirits.

Thank you for your prompt response and valuable feedback on our manuscript submitted to The Plant Cell. On behalf of all the contributing authors, I would like to express our sincere appreciations of your constructive comments concerning our article entitled "**Evolutionary amplification and functional divergence of UDP-dependent glycosyltransferases genes shapes the quality traits and cold tolerance of tea plants" (Manuscript NO: TPC2024-RA-00024D)**. We sincerely thank you for your valuable suggestions on our manuscript. Based on these suggestions, we have carefully revised the manuscript and the detailed revisions in the manuscript are given in the red text and are listed below.

Reviewer #1:

1. The authors have made extensive revisions to the manuscript, significantly improving its clarity and the reliability of the data.

Response: Thank you very much for your feedback on our revised manuscript. We greatly value your comments, and your suggestions have greatly helped us improve our work.

Reviewer #2

Major comments:

1. In my previous review, I had asked the authors to demonstrate that AsODNs were specifically targeting the desired genes, and it appears that they have provided these important controls in the revised manuscript.

Response: Thank you for your careful review and feedback. Your suggestion has been invaluable in improving our work.

2. In my previous review, I asked to see gels of the purified glycosyltransferases. These gels have been provided in the revision. None of these proteins are actually pure and the authors are assuming that they have produced the correct protein simply

by estimating the molecular weight of each recombinant protein on SDS-PAGE gels. These observations are problems for a few reasons: 1) the authors refer to these proteins throughout the manuscript as "purified", and they are not, 2) any effort to standardize these recombinant proteins on a protein mass basis (i.e. 3 micrograms of protein per reaction, etc) is not valid because the proteins are not pure, and 3) because many of the purification gels look similar, it is hard to discern whether some of the measured activities arise from these GTs or E. coli GTs that are consistently co-purifying. To be confident that the ascribed proteins are contributing to the ascribed activities, the authors need to purify these proteins to homogeneity. This could readily be accomplished by performing a secondary anion exchange or size exclusion purification on an FPLC or installing a his-tag on the previous recombinant proteins to perform Glutathione- nickel NTA sequential purifications. Minimally, the authors could only refer to these proteins as partially purified throughout the manuscript and perform mass spectrometry analysis of dissected bands from the SDS-PAGE gel to show that the indicated bands are actually the GT of interest.

Response: We sincerely thank you for your valuable suggestions. According to your suggestions, we isolated and purified the recombinant protein using ion-exchange chromatography and obtained the pure target protein. In addition, we re-performed a substrate screening of the purified proteins. Interestingly, the optimal substrate for glycosylation of UGTs was not changed, except for minor changes in some of the substrates. The details are as listed below.

Line 615-624: The obtained recombinant proteins were loaded onto a HiTrap Q XL anion-exchange column (Cytiva) for size exclusion purification by fast protein liquid chromatography (FPLC, BIO-RAD). After washing with 10 mL of buffer A (20 mM Tris, pH 8.0, 20 mM NaCl), the column was eluted with a 20 mL gradient of 20 to 1000 mM NaCl at a flow rate of 0.8 mL/min, and the collected fractions were identified by SDS - polyacrylamide gel electrophoresis (SDS-PAGE) (Supplementary Fig. S15). The target proteins were concentrated and recovered using Amicon Ultra filters (Merck), the purified proteins concentration was calculated according to the standard curve (Supplementary Fig. S16). Finally, the purified proteins were stored at

-80 ℃ for subsequent analysis.

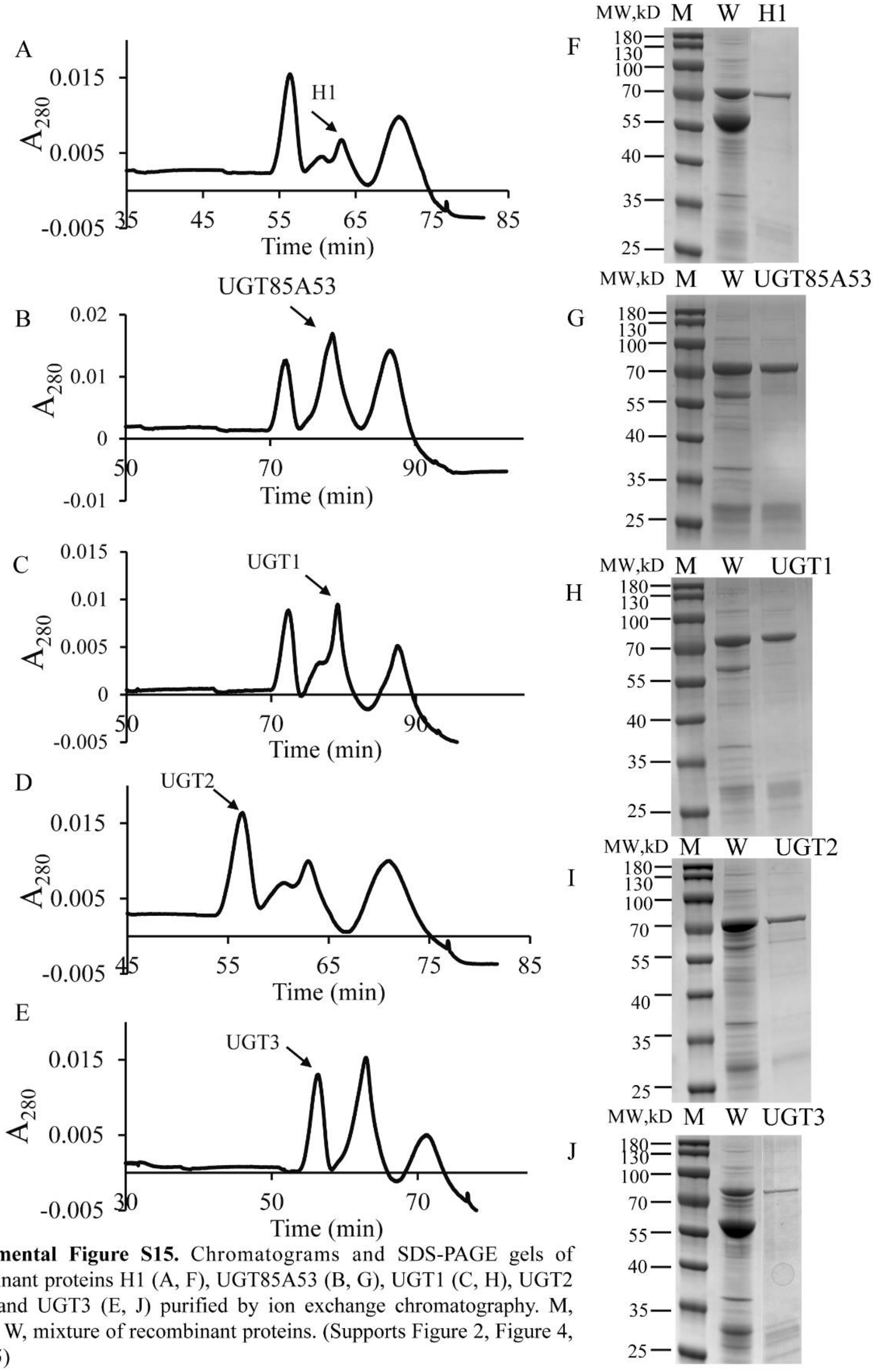

Supplemental Figure S15. Chromatograms and SDS-PAGE gels of recombinant proteins H1 (A, F), UGT85A53 (B, G), UGT1 (C, H), UGT2 (D, I), and UGT3 (E, J) purified by ion exchange chromatography. M, marker; W, mixture of recombinant proteins. (Supports Figure 2, Figure 4, Figure 5)

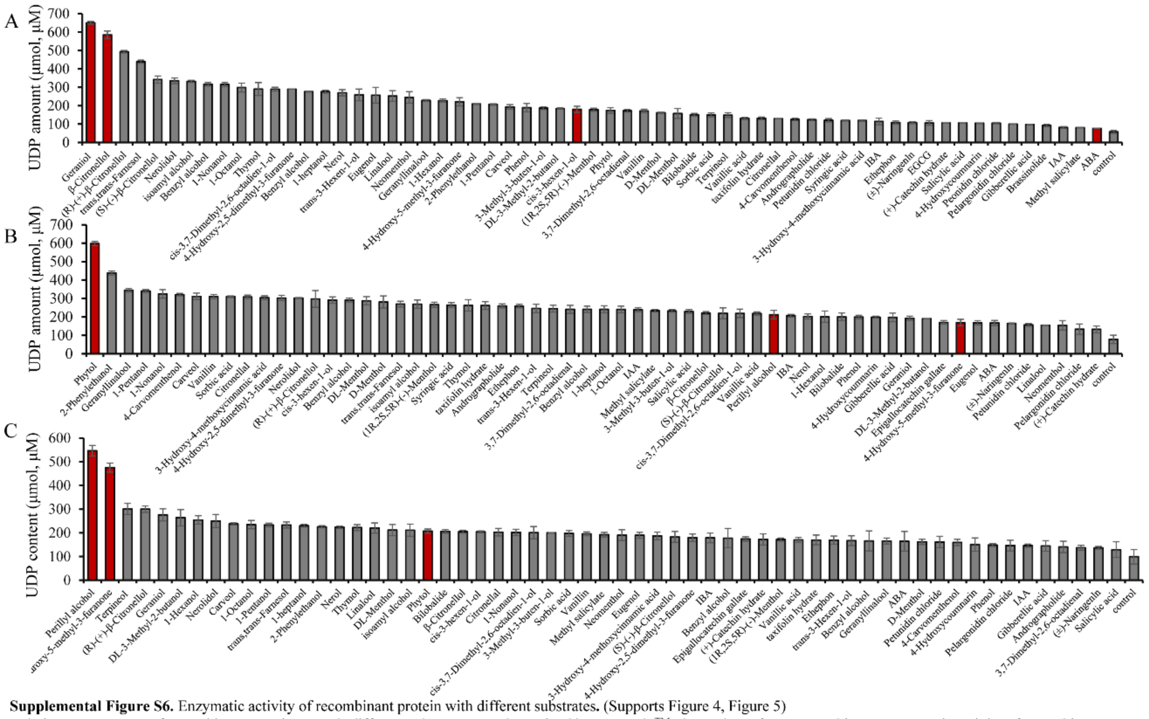

Supplemental Figure S6. Enzymatic activity of recombinant protein with different substrates**.** (Supports Figure 4, Figure 5)
Relative UDP content of recombinant protein towards different substrates was determined by UDP-Glo™ glycosyltransferase assay kit. **(A)** Enzymatic activity of recombinant UGT1 with different substrates. Substrates compared to UGT85A53 are labeled in red. **(B)** Enzymatic activity of recombinant UGT3 with different substrates. Substrates compared to UGT2 are labeled in red. **(C)** Enzymatic activity of recombinant UGT2 with different substrates. Substrates compared to UGT3 are labeled in red. Relative amounts to UDP calculated from standard curves, and each reaction contained at least three biological replicates

We tried our best to improve the manuscript and made some changes marked in red in revised paper which will not influence the content and framework of the paper. We appreciate for your warm work earnestly, and hope the correction will meet with approval. Once again, thank you very much for your comments and suggestions. If you have any queries, please don't hesitate to contact me (sckfriend@163.com, xiaenhua@gmail.com, Wilfried.Schwab@tum.de).

Yours sincerely,

Chuankui Song, Enhua Xia, and Wilfried Schwab

1st Revision - Editorial Decision11-Aug-2024

Dear Dr. Chuankui Song:

We have received reviews of your manuscript entitled "Evolutionary amplification and functional divergence of UDP-dependent glycosyltransferases genes shapes the quality traits and cold tolerance of tea plants." On the basis of the advice received, the board of reviewing editors would like to accept your manuscript for publication in The Plant Cell. This acceptance is contingent on revision based on the comments of our reviewers. In particular, please consider the following:

As you will see below, Reviewer #1 has identified 3 final points that require correction (point #1) and clarification (points #2 and #3). Please check Fig. S3 and S15 to make sure you are presenting the intended data. For Fig. S3, you may want to highlight peaks of interest and describe the control in the caption.

A note from the Editor-in-Chief: Please revised your figures and supplemental figures so that they all use a "sans serif" font, preferably Arial, as described in our Instructions to Authors.
In addition, we don't typically publish political maps as used in Figure 1, because of controversies over borders. I suggest that you either switch this to a small descriptive table (which could be supplemental) listing the provinces and locations (and readers can look them up if they have any questions), or use a physical map that lacks international borders, and you could overlay only the subset of provincial borders that are relevant. The former option may be easier than the latter.

I look forward to seeing the final revision for this manuscript. To submit your revised manuscript, click:

Link Not Available

If you have any questions about the revision submission procedures, please contact the Editorial Office Staff (tpc-submissions@aspb.org). If you cannot return the revised manuscript within 30 days, please let us know. Otherwise, we will assume that you have elected not to revise the manuscript and withdraw it.

Thank you very much for the privilege of reviewing this work. I look forward to receiving the next version.

On behalf of the editorial board,

James Leebens-Mack, Board of Reviewing Editors
Daniel Kliebenstein, Senior Editor
Blake Meyers, Editor-in-Chief

The Plant Cell

Reviewer #1 (Comments for the Author):

The authors have made significant efforts to purify the UGT proteins, and the results appear satisfactory. Importantly, the conclusions remain unchanged with the additional purification. However, I have two concerns regarding the newly added information:

1. The statement "The obtained recombinant proteins were loaded onto a HiTrap Q XL anion-exchange column (Cytiva) for size exclusion purification" is incorrect. An anion-exchange column is used for anion-exchange purification, not size exclusion purification.

2. Regarding the anion-exchange chromatography (Supplementary Fig. S15): It is somewhat surprising that the chromatography profiles of H1(A) and UGT2(D) are so similar, given that their protein mixtures appear different as shown by the SDS-PAGE (F and I). Additionally, both the chromatography (B and C) and SDS-PAGE (G and H) profiles for UGT85A53 and UGT1 are very similar, which differs from the data provided in the previous revision. It may be necessary to confirm the reliability of these data to rule out the possibility of sample or data confusion. Moreover, the conductivity curve should be provided in the anion-exchange chromatography, and the y-axis unit is missing.

3. Beyond the added information, I also have a concern regarding Supplementary Fig. S3. The figures presented do not appear to be LC-MS/MS results. The meaning of the label "control" is unclear, and it is difficult to discern any peak signals in the figures.

Reviewer #2 (Comments for the Author):

In previous reviews of this manuscript, I had asked the authors to provide gels of the recombinant enzymes that they produced

to examine substrate preferences of these target proteins. This information was provided in a previous resubmission, but the recombinant proteins were not pure. This is an important issue when characterizing the substrate specificity of recombinant proteins. In this resubmission, the authors have purified each glycosyltransferase to homogeneity and demonstrated that the activities that they initially observed are consistent when repeated with purified proteins.

I am now more confident in these results, and I do not have further comments that need to be addressed.

Please also note the following:

-The Plant Cell now requires authors to complete and submit an author revisions checklist upon submission of a revised manuscript. The aim of the checklist is to aid authors in preparing a high-quality manuscript, facilitate the review and assessment of revised manuscripts, and help to ensure that journal standards are maintained across the board. If your manuscript is accepted, the completed checklist will be published as supplemental material attached to the article online. Please download a copy of the checklist (pdf fillable form) at this link, for submission with your revised manuscript: https://tpc.msubmit.net/html/Author_Revisions_Checklist.pdf.

-Supplemental materials should be restricted to large datasets and tables, presentation of replicates, and validation of reagents, methods, or genotypes. Any data that are used to support the major claims must be in the main manuscript. Supplemental figure legends must indicate what figure in the main manuscript is supported by the supplemental data presented. Please justify how each of the supplemental figures meet the criteria.

-Sampling methods and the nature of "biological replicates" should be described precisely (i.e. different plants, parts of plants, pooled tissue, independent pools of tissue, sampled at different times, etc.), along with a clear description of and rationale for any statistical analyses conducted. The reader should know exactly what was sampled; what forms the basis of the calculation of any means and statistical parameters reported. This is also necessary to ensure that proper statistical analysis was conducted.

Dear Editor and Reviewers,

I hope this letter finds you in good health and high spirits. We would like to thank you for the positive and constructive comments and suggestions on our manuscript submitted to The Plant Cell. On behalf of all the contributing authors, I would like to express our sincere appreciations of your constructive comments concerning our article entitled "**Evolutionary amplification and functional divergence of UDP-dependent glycosyltransferases genes shapes the quality traits and cold tolerance of tea plants" (Manuscript NO: TPC2024-RA-11127D)**. We sincerely thank you for your valuable suggestions on our manuscript. Based on these suggestions, we have carefully revised the manuscript and the detailed revisions in the manuscript are given in the red text and are listed below.

Reviewer #1:

1. The statement "The obtained recombinant proteins were loaded onto a HiTrap Q XL anion-exchange column (Cytiva) for size exclusion purification" is incorrect. An anion-exchange column is used for anion-exchange purification, not size exclusion purification.

Response: We sincerely thank you for your valuable suggestions. We have corrected this error based on your suggestion. The details are as listed below.

Line 608-610: The obtained recombinant proteins were loaded onto a HiTrap Q XL anion-exchange column (Cytiva) for anion-exchange purification by fast protein liquid chromatography (FPLC, BIO-RAD).

2. Regarding the anion-exchange chromatography (Supplementary Fig. S15): It is somewhat surprising that the chromatography profiles of H1(A) and UGT2(D) are so similar, given that their protein mixtures appear different as shown by the SDS-PAGE (F and I). Additionally, both the chromatography (B and C) and SDS-PAGE (G and H) profiles for UGT85A53 and UGT1 are very similar, which differs from the data provided in the previous revision. It may be necessary to confirm the reliability of these data to rule out the possibility of sample or data confusion. Moreover, the

conductivity curve should be provided in the anion-exchange chromatography, and the y-axis unit is missing.

Response: Many thanks for your constrictive suggestions. We performed anion-exchange purification for each UGT separately, we are sure that there is no sample or data confusion. As for the phenomenon that the chromatograms and SDS-PAGE gels are similar, it may be because these UGTs belong to the same subfamily and the molecular weights of the proteins are closer to each other. In addition, we added conductivity curves in the anion-exchange chromatography. The details are as listed below.

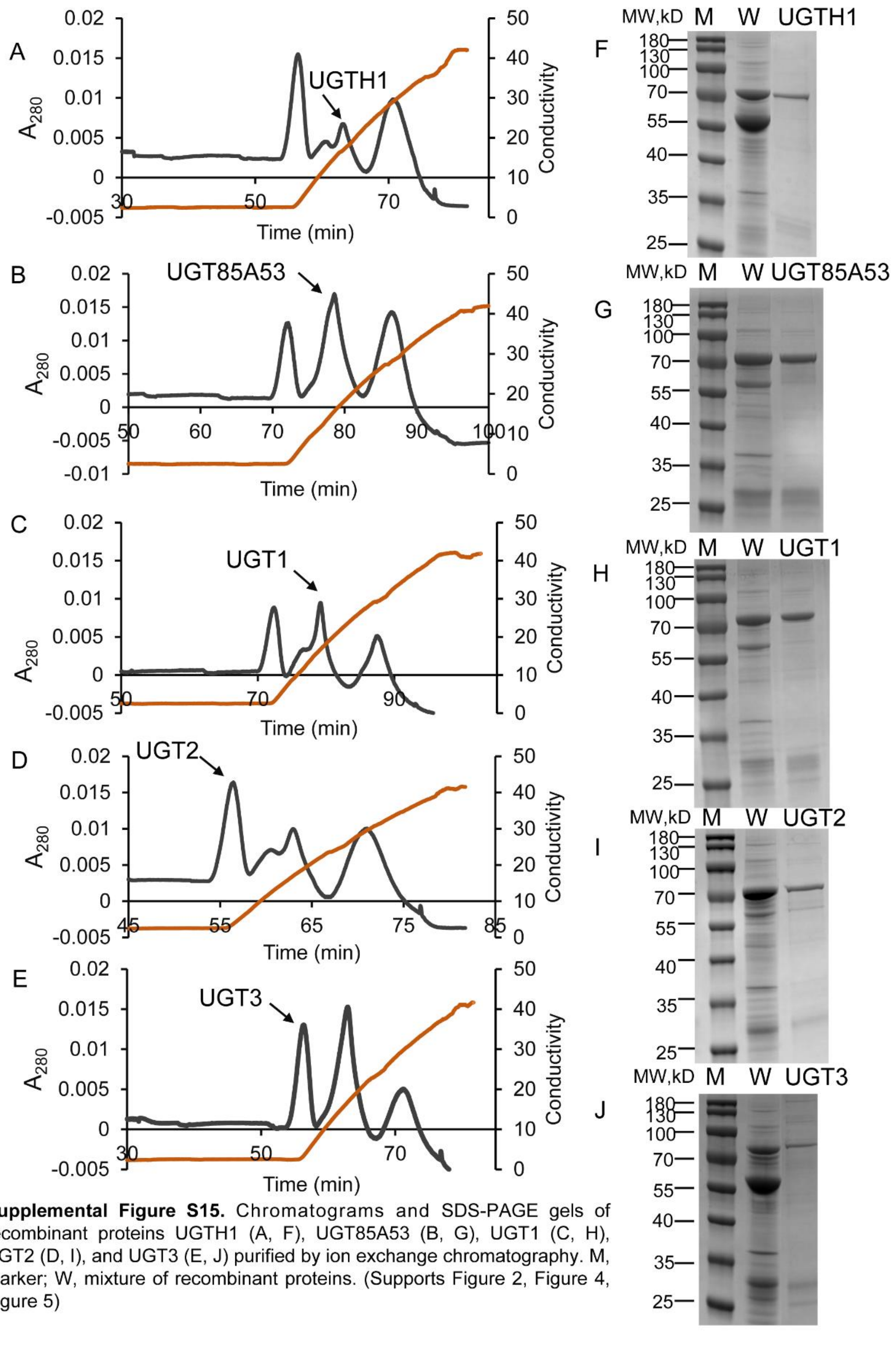

Supplemental Figure S15. Chromatograms and SDS-PAGE gels of recombinant proteins UGTH1 (A, F), UGT85A53 (B, G), UGT1 (C, H), UGT2 (D, I), and UGT3 (E, J) purified by ion exchange chromatography. M, marker; W, mixture of recombinant proteins. (Supports Figure 2, Figure 4, Figure 5)

3. Beyond the added information, I also have a concern regarding Supplementary Fig. S3. The figures presented do not appear to be LC-MS/MS results. The meaning of the label "control" is unclear, and it is difficult to discern any peak signals in the figures.

Response: Thank you for your suggestion. The Supplementary Fig. S3 indicates that H1 has no glycosyltransferase activity for SA and NHP, so there is no peak signal. In order to express this result more directly, we modified the figure caption and indicated the meaning of the label "control". The details are as listed below.

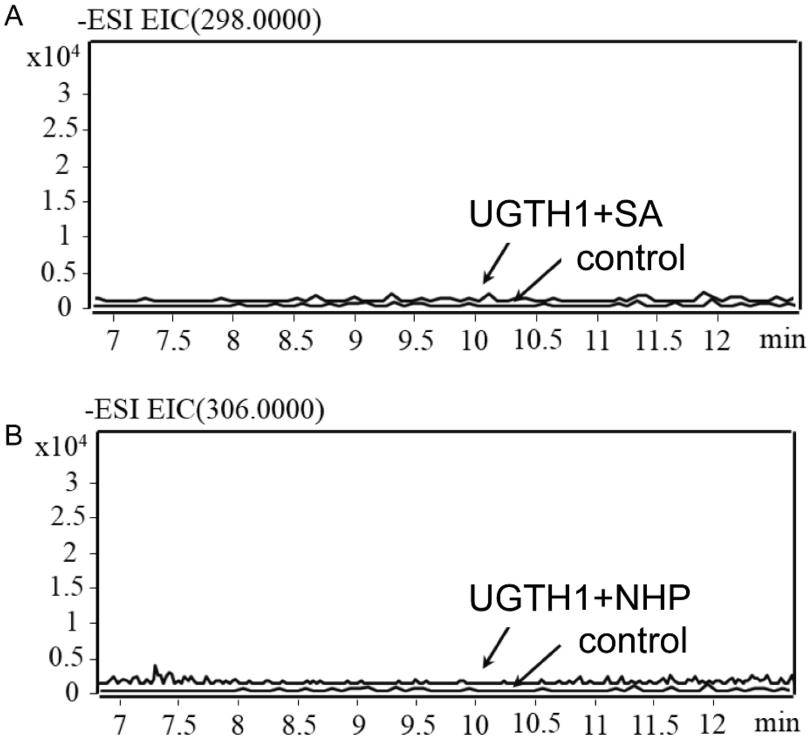

Supplemental Figure S3. LC–MS/MS analyses of enzymatic products formed by UGTH1**.** (Supports Figure 2)
(A) Recombinant UGTH1 has no glycosyltransferase activity to salicylic acid (SA).
(B) Recombinant UGTH1 has no glycosyltransferase activity to N-Hydroxypipecolic acid (NHP).
'control' represents a reaction system containing an equal amount of empty vector protein.

We tried our best to improve the manuscript and made some changes marked in red in revised paper which will not influence the content and framework of the paper. We appreciate for your warm work earnestly, and hope the correction will meet with

approval. Once again, thank you very much for your comments and suggestions. If you have any queries, please don’t hesitate to contact me (sckfriend@163.com, xiaenhua@gmail.com, Wilfried.Schwab@tum.de).

Yours sincerely,

Chuankui Song, Enhua Xia, and Wilfried Schwab

Dear Dr. Chuankui Song:

We are pleased to inform you that your paper entitled "Evolutionary amplification and functional divergence of UDP-dependent glycosyltransferases genes shapes the quality traits and cold tolerance of tea plants" has been accepted for publication in The Plant Cell, pending a final editorial review by a science editor. At this stage, your manuscript will be evaluated by a science editor with respect to its presentation of scientific content, compliance with journal policies, and presentation for a broad readership. The Plant Cell has contracted with Plant Editors (planteditors.com) to provide this service to our authors, and you will soon receive additional information on this process.

As you may have seen, we will be publishing a Focus Issue on Phylogenomics next summer. This work very nicely shows how how phylogenetic analyses of gene families can powerfully motivate experimental investigation of shifts in gene function, and help frame the evolutionary implications of experimental results. Your manuscript can be published before next summer and added to the Phylogenomics Focus Issue web page when it is launched.

Please note that each author needs to link their ORCID identifier to their account in the system before your manuscript can be published. If any authors do not have an ORCID linked to their account, they will receive a message with a link to complete this task. Please ensure that ALL of your coauthors have completed this task as soon as possible.

LICENSE INFORMATION Shortly after your paper is sent to production, you will receive an email from our publisher's author support team, **SciPris**, with information on article processing charges, as well as the types of Open Access license you would be able to purchase. The accepted version of your manuscript will not appear on our Advance Articles page until the license is selected and signed, so please look out for an email from SciPris. For more information see the Article Publication Fees section of our author guidelines: https://academic.oup.com/plcell/pages/General-Instructions

The Plant Cell and The Arabidopsis Information Resource (TAIR) are collaborating to collect functional annotation data about Arabidopsis genes from authors. This includes information about the gene's molecular function (e.g., kinase activity, ATP synthetase activity), the biological process/es it is involved in (e.g., endosperm development, threonine biosynthesis), its subcellular location (e.g., nucleus, ER), anatomical or developmental expression pattern (e.g., leaf, ovule, flower stage 10, seedling stage), or its partner in a protein-protein interaction (e.g., AT1G01010 interacts with AT1G01020).

If your paper contains results falling into one or more of these categories for Arabidopsis genes, we request that you now submit these data for inclusion in TAIR by filling in the form provided at the following URL: https://www.arabidopsis.org/doc/submit/functional_annotation/123. If you need further clarification on what types of data can be submitted please contact curator@arabidopsis.org.

Finally, we encourage your submission of artwork for the journal cover. If there is an image or illustration related to your paper that you would like to have considered, please email it in .pdf or .tif format to clowe@aspb.org. An image title, brief, 2-3 sentence description of the image, and the name of the person credited for the image are also encouraged. If your image is selected to appear on the online cover of the journal, you will be contacted with the scheduled issue date and further information.

We look forward to seeing your paper published.

On behalf of the editorial board,

James Leebens-Mack, Board of Reviewing Editors
Daniel Kliebenstein, Senior Editor
Blake Meyers, Editor-in-Chief

The Plant Cell

=====
IMPORTANT REMINDER: PEER REVIEW REPORTS
=====

If you opted to publish a peer review report along with your article during the original submission process, it will be prepared by the editorial staff and publicly posted with your manuscript, inside the zip file that contains any other supplemental material. As a reminder, the peer review report is a public record of all comments from editors and reviewers, as well as your prior responses, as you received them in the decision letters for each draft of your manuscript. If you agreed to publish this report and have changed your mind, or are not sure if you selected this option, please contact the editorial office as soon as possible before signing the license agreement from our publisher.

=====
REVIEWER COMMENTS
=====

----FOR ASPB OFFICE USE ONLY (DO NOT EDIT)----
MSID: 40524
Corresponding Author: Prof. Dr. Song
